# Identification and characterization of mixed infections of *Chlamydia trachomatis via* high-throughput sequencing

**DOI:** 10.3389/fmicb.2022.1041789

**Published:** 2022-11-10

**Authors:** Jianhui Zhao, Jingwei Shui, Lipei Luo, Cailing Ao, Hongqing Lin, Yuanhao Liang, Li Wang, Haiying Wang, Hongliang Chen, Shixing Tang

**Affiliations:** ^1^Department of Epidemiology, School of Public Health, Southern Medical University, Guangzhou, China; ^2^Department of Clinical Microbiology Laboratory, Chenzhou No. 1 People’s Hospital, Chenzhou, China

**Keywords:** *Chlamydia trachomatis*, next-generation high-throughput sequencing, genotype, mixed-genotype infection, clinical manifestation

## Abstract

Precise genotyping is necessary to understand epidemiology and clinical manifestations of *Chlamydia trachomatis* infection with different genotypes. Next-generation high-throughput sequencing (NGHTS) has opened new frontiers in microbial genotyping, but has been clinically characterized in only a few settings. This study aimed to determine *C. trachomatis* genotypes in particular mixed-genotype infections and their association with clinical manifestations and to characterize the sensitivity and accuracy of NGHTS. Cervical specimens were collected from 8,087 subjects from physical examination center (PEC), assisted reproductive technology center (ART) and gynecology clinics (GC) of Chenzhou Hospital of China. The overall prevalence of *C. trachomatis* was 3.8% (311/8087) whereas a prevalence of 2.8, 3.7 and 4.8% was found in PEC, ART and GC, respectively. The most frequent three *C. trachomatis* genotypes were E (27.4%, 83/303), F (21.5%, 65/303) and J (18.2%, 55/303). Moreover, NGHTS identified 20 (6.6%, 20/303) mixed-genotype infections of *C. trachomatis*. Genotype G was more often observed in the subjects with pelvic inflammatory disease than genotype E (adjusted *OR* = 3.61, 95%*CI*, 1.02–12.8, *p* = 0.046). Mixed-genotype infection was associated with severe vaginal cleanliness (degree IV) with an adjusted *OR* of 5.17 (95%*CI* 1.03–25.9, *p* = 0.046) whereas mixed-genotype infection with large proportion of minor genotypes was associated with cervical squamous intraepithelial lesion (SIL) with an adjusted *OR* of 5.51 (95%*CI* 1.17–26.01, *p* = 0.031). Our results indicated that NGHTS is a feasible tool to identity *C. trachomatis* mixed-genotype infections, which may be associated with worse vaginal cleanliness and cervical SIL.

## Introduction

*Chlamydia trachomatis* is one of the most widespread sexually transmitted diseases (Fu et al., 2022). Approximately, 80% of *C. trachomatis* infections are essentially asymptomatic (Marcone et al., 2012). Persistent *C. trachomatis* infection can cause various sequelae such as urethritis, endometritis, pelvic inflammatory disease (PID), tubal factor infertility, and ectopic pregnancy (Woodhall et al., 2018). *C. trachomatis* includes 19 genotypes (Bax et al., 2011). In general, genotype A, B and C are usually associated with trachoma while genotype D-K primarily cause urogenital infection (Bax et al., 2011). In addition, genotype L1-L3 are the agents of lymphogranuloma venereum (Jurstrand et al., 2001).

The *ompA* is one of the most variable genes in the *C. trachomatis* genomes and encodes the main outer membrane protein (MOMP) (Nunes et al., 2009). The *ompA* gene contains four highly polymorphic variable sequences VS1–4, which are separated by five constant sequences CS1–5 (Spaargaren et al., 2005). The most variable and discriminatory nucleotide sequences are found in the VS1 and VS2 regions, which make them the suitable target fragments for *C. trachomatis* genotyping (Spaargaren et al., 2005). However, previous studies showed that the recombination and horizontal gene transfer of *ompA* is a natural phenomenon occurring within some *C. trachomatis* strains (Somboonna et al., 2011; Harris et al., 2012; Matičič et al., 2016). Although the *ompA* gene of *C. trachomatis* is a single copy gene and may have a lower probability of switching compared to the multi-copy genes, e.g., cryptic plasmid (Joseph et al., 2011), it may not always represent the genetic background of the *C. trachomatis*.

At present, the methods for *C. trachomatis* genotyping include Sanger sequencing, polymerase chain reaction-based restriction fragment length polymorphism (PCR-RFLP) (Petrovay et al., 2015; Foschi et al., 2016), hybridization methods (Ruettger et al., 2011; Gharsallah et al., 2012; Martinez et al., 2015; Brasiliense et al., 2016), real-time PCR using fluorescent probes (Jalal et al., 2007), DNA microarray assay (Gallo Vaulet et al., 2016) and whole genome sequencing (WGS; Brown and Christiansen, 2019). Sanger sequencing is the most widely used technique for genotyping, but is not adequate to detect mixed-genotype infections of *C. trachomatis* since it only provides one consensus sequence (Quint et al., 2007; Ruettger et al., 2011; Gharsallah et al., 2012). Although PCR-RFLP, hybridization methods and DNA microarray assay can identify mixed-genotype infections to some extent (Ruettger et al., 2011; Gharsallah et al., 2018), their sensitivity and reliability remain to be improved. In contrast, next-generation high-throughput sequencing (NGHTS) targeting variable regions of *ompA* gene can not only inherit the advantage of *ompA* Sanger sequencing to identify *C. trachomatis* genotypes, but also determine mixed-genotype infections and the proportion of different genotypes in the mixed-genotype infections due to enough sequencing depth and large number of sequencing reads (Kawada et al., 2016), which are more suitable for the detection of co-infections or super-infections of different *C. trachomatis* genotypes.

Indeed, the previous studies of *C. trachomatis*-infected patients or animals have demonstrated the difference of *C. trachomatis* genotypes in virulence and pathogenicity (Ito et al., 1990; Lyons et al., 2004). However, these studies only compared the virulence of different *C. trachomatis* genotypes (Batteiger et al., 1989; Workowski et al., 1994; Ngandjio et al., 2003; Lyons et al., 2004). It remains to elucidate whether mixed-genotype infection of *C. trachomatis* could result in enhanced virulence and may account for divergent clinical outcomes of *C. trachomatis* infection. Few studies have attempted to correlate specific clinical manifestations of genital mixed-genotype infection of *C. trachomatis* in humans because of the technical difficulty to identify mixed-genotype infections (Yan et al., 2018).

In this study, we explored the feasibility of NGHTS to determine *C. trachomatis* genotypes and to identify mixed-genotype infections in a large number of *C. trachomatis-*positive cervical samples in a cross-sectional and observational study. The objective of this study was to characterize the sensitivity and accuracy of NGHTS and to determine *C. trachomatis* genotypes in particular mixed-genotype infections and their association with clinical manifestations.

## Materials and methods

### Study participants and clinical samples

A total of 8,087 samples of cervical swabs from the physical examination center (PEC), assisted reproductive technology center (ART) and gynecology clinics (GC) of Chenzhou No.1 People’s Hospital in Chenzhou of Hunan Province, China were randomly collected and tested for *C. trachomatis* nucleic acid from March 1, 2019 to July 13, 2021. The hospital is the biggest general hospital in Chenzhou and consists of five campuses to cover the whole city and nearby regions. The 2,950 female subjects from PEC were those for routine annual health examinations while the 1,666 female subjects from ART and 3,471 female subjects from GC were those for diagnosis and treatment of infertility and gynecological diseases, respectively. The inclusion criteria were female, and not pregnant. Cervical swab samples were collected using a 200 mm polyethylene Cervix brush device (Hybribio Corp, Guangzhou, China). The specimens were transferred to a tube containing cervical cell preservation solution provided in the kit and stored at −80°C until analysis. For *C. trachomatis* positive subjects, demographic characteristics, antibiotic usage during the previous 3 months, human papillomavirus (HPV) infection, clinical symptoms, vagina cleanliness, cervical abnormalities were retrospectively collected. To detect HPV infection, DNA was extracted from cervical swab samples within 48 h after collection using the QIAamp mini kit (Qiagen, Hilden, Germany). HPV detection and genotyping were performed by using the Hybribio Rapid Geno-Array test kit (Hybribio Corp, Guangdong) based on the PCR-reverse dot blot hybridization method. The study was conducted in Chenzhou No.1 People’s Hospital, China, under the Principles of the Declaration of Helsinki, and was approved by the Ethical Committee of Chenzhou No.1 People’s Hospital (CZ/1128). Written informed consent was obtained from all the participants. All the experiments were carried out in the lab certified by the National Center for Clinical Laboratories following the laboratory biosafety guidelines (Burnett et al., 2009).

### Clinical manifestations and diagnosis

Asymptomatic *C. trachomatis* infection was defined as positive for *C. trachomatis* nucleic acid without symptoms, such as painful sexual intercourse, abnormal vaginal discharge, urethritis, irregular vaginal bleeding, or bleeding after sexual intercourse and genital warts (Chen et al., 2020). Vaginosis was diagnosed according to Amsel criteria (Carr et al., 1998). There are many methods to evaluate the vaginal microenvironment. In China, the vaginal cleanliness grade is also used to comprehensively evaluate the status of the vaginal microenvironment (Group., C.M.A.O.a.G.B.I.D.C, 2016), and has been widely accepted for gynecological studies (Yue et al., 2015; Yu et al., 2018). Vaginal cleanliness is classified as I, II, III and IV grades according to bacterium vaginae, Coccus, epithelial cell and leukocytes (Supplementary Table S1). Class I and II are considered normal while class III and IV as abnormal (Bao et al., 2015), and Grade IV vaginal cleanliness is regarded as severe vaginal cleanliness. The vaginal cleanliness is characterized by microscopy and bacterial morphology, and cannot identify the specific species of bacterium vaginae. PID is defined as tenderness with adnexal, cervical motion, and uterine tenderness (Dean et al., 1995). Cervicitis is determined by evaluating and scoring the clinical findings at the time of speculum examination. A score of ≥3 is defined as cervicitis while ≤2 as no cervicitis (Supplementary Table S1; Batteiger et al., 1989). Colposcopy screening is performed using a digital electronic colposcopy (SLC-3000, Philips, Shenzhen, China) following a standard procedure (Chen et al., 2020). According to the standard and terminology of the American Society for Colposcopy and Cervical Pathology (ASCCP) (Khan et al., 2017), colposcopy impression includes benign, low-grade squamous intraepithelial lesion (LSIL), high-grade squamous intraepithelial lesion (HSIL), and cancer. LSIL and HSIL represent Grade 1 (minor) and Grade 2 (major) abnormal colposcopy findings defined by the International Federation for Cervical Pathology and Colposcopy (IFCPC) nomenclature, respectively (Bornstein et al., 2012). The samples for cytology analysis are harvested by using polyethylene cervix brush device and cervical cell preservation solution (Hybribio Corp, Guangzhou, China). Sectioning and staining are conducted in all-in-one machine (Dacheng, Guangzhou, China). The cytology results are plotted in a table and categorized according to the Bethesda system (TBS) (de Oliveira et al., 2020). The following variables are considered: no malignancy, ASC-US, LSIL, ASC-H, HSIL, squamous cell carcinoma, atypical glandular cells (AGC), adenocarcinoma, and other malignant neoplasms.

### Detection and genotyping of *Chlamydia trachomatis* by PCR and sanger sequencing

DNA was extracted from the cervical swabs using QIAamp DNA Minikit QIAgen (Qiagen, Hilden, Germany) according to the manufacturer’s instructions. The isolated DNA was stored at −80°C until use for PCR and sequencing. A 200 bp conserved cryptic plasmid Pgp2 fragment of *C. trachomatis* was amplified by PCR for diagnosis of *C. trachomatis* infection with the primers of CT-d-F and CT-d-R (Supplementary Table S2). PCR was carried out in 25 μl reaction mixture in a thermal cycler with the following reaction conditions: 95°C for 2 min, followed by 35 cycles of 95°C for 15 s, 55°C for 30 s and 72°C for 40 s with a final elongation at 72°C for 5 min.

For genotyping, *C. trachomatis ompA* fragment VS1–VS4 was first amplified by nested PCR using the outer primers CT1 and CT2 followed by the amplification of a 580 bp VS1–VS2 fragment using the inner primers CT3 and CT4. First round PCR was carried out in 25 μl reaction mixture with the following reaction conditions: 95°C for 5 min, followed by 25 cycles of 95°C for 60 s, 55°C for 60 s and 72°C for 80 s, with a final elongation at 72°C for 10 min. The reaction conditions of the second round PCR were: 95°C for 5 min, followed by 35 cycles of 95°C for 30 s, 55°C for 30 s and 72°C for 30 s, with a final elongation at 72°C for 10 min. All the primer sequences were listed in the Supplementary Table S2. The *C. trachomatis* strain (ATCC VR-348B) was used as positive control and DNase-free water as negative control in PCR. PCR products for *C. trachomatis ompA* gene were sent out for Sanger sequencing in Ruibo Biotech (Guangzhou, China). Genotypes of *C. trachomatis* were determined by BLAST as previously described (Yang et al., 2010).

### Identification of mixed-genotype infections of *Chlamydia trachomatis* using NGHTS

For *C. trachomatis* genotyping through NGHTS, a 448 bp fragment of *C. trachomatis ompA* gene VS1–VS2 region was amplified using nested PCR with the following primers (Supplementary Figure S1): outer primes *ompA* CT-HTS-F-outer/CT-HTS-R-outer and inner primers CT-HTS-F-inner/CT-HTS-R-inner with barcode (Supplementary Table S2). The first round PCR was carried out in 25 μl reaction mixture, with 12.5 μl of Phanta^®^ Max Super-Fidelity DNA polymerase (Vazyme Biotech, Nanjing, China), 0.5 μl of forward and reverse primers (10 pmol/ul), and the following reaction conditions: 95°C for 3 min, followed by 25 cycles of 95°C for 15 s, 55°C for 30 s and 72°C for 40 s, with a final elongation at 72°C for 5 min. The second round PCR was carried out in 50 μl reaction volume with 25 μl of Phanta^®^ Max Super-Fidelity DNA polymerase (Vazyme Biotech, Nanjing, China), 1 μl of forward and reverse primers (10 pmol/ul), and 2 μl first-round PCR product. Thermal cycling consisted of initial denaturation at 95°C for 3 min, followed by 35 cycles of denaturation at 95°C for 15 s, annealing at 55°C for 15 s, and elongation at 72°C for 30 s with final incubation at 72°C for 5 min. The second-round PCR products were purified using universal DNA Purification Kit (TIANGEN Biotech, Beijing, China) according to the manufacturer’s manual, and quantified using GENOVA NANO (Bibby Scientific Ltd., Stone, United kingdom). Finally, 10 samples were mixed at 1 μg of purified DNA per sample and confirmed by electrophoresis on 2% agarose gel.

Sequencing libraries were generated using NEB Next^®^ UltraTM DNA Library Prep Kit for Illumina (NEB, Massachusetts, United States) following the manufacturer’s recommendations and index codes were added. The library quality was assessed on the Qubit@ 2.0 Fluorometer (Thermo Scientific, Massachusetts, United States) and Agilent Bioanalyzer 2100 system (Agilent Technologies, CA, United States). Finally, the library was sequenced on an Illumina NovaSeq 6000 and 250 bp paired-end reads were generated. To make the analysis results more accurate and reliable, the original data were first spliced and filtered to obtain clean data. The clean data was obtained using fastp software, with the following criterion (Novogene Technology Co., LTD, Beijing, China): when the N in any sequencing read exceeds 10% of the total number of reads, or the number of bases with low quality (Q ≤ 5) in any sequencing reads exceeds 50% of the total number of reads, or sequencing read contains adapter sequences, these reads are eliminated. Paired-end reads from the original DNA fragments were merged by using the FLASH program, which inputs a fastq library of paired-end reads (reads1 and reads2) in which some of the reads overlap the read generated from the opposite end of the same DNA fragment, and merged the fragments based on the correct overlap between the paired-end reads (Magoč and Salzberg, 2011). The sequences of different samples were extracted based on the specific barcode sequences. Burrows-Wheeler transform (BWA 0.7.17; Li and Durbin, 2009) with the default parameters was used for aligning all the clean sequence data with the reference sequences of *C. trachomatis* genotype A-K and L1-L3. Based on the results of sequence alignment, the genotype of each read was determined and the composition of different genotypes was calculated. The proportion of minor genotype >1% is defined as a mixed-genotype infection (Quer et al., 2015). The genotype with the large proportion is considered to be the major genotype in the case of a mixed-genotype infection identified. The reference sequences used in this study included A/Sa1(M58938), B/ IU1226 (AF063208), C/TW3 (M17343), D/ UW3 (AE001338), E/Bour (X52557), F/IC-Cal3 (X52080), G/UW57 (AF063199), H/UW4 (X16007), I/UW-12 (AF063200), J/UW36 (AF063202), K/UW31 (AF063204), L1/440 (M36533), L2/434 (M14738), and L3/404 (X55700).

### Detection limit of NGHTS for identifying mixed genotypes

A 456 bp fragment of the *ompA* gene was amplified from the clinical samples infected with *C. trachomatis* genotype B, D, E, F, G, H, J and K, and cloned into the pUC57 vector (TsingKe Biotech Corp, Beijing, China). The plasmid DNA was purified and quantified using a GENOVA NANO (Bibby Scientific Ltd., Stone, United Kingdom). The DNA copy number was calculated using the following formula: DNA copy number (copy number/μL) = [6.02 × 10^23^ × plasmid concentration (ng/μL) × 10^−9^]/[DNA in length × 660]. A serial 10-fold diluted plasmid DNAs for *C. trachomatis* genotype B, D, E, F, G, H, J and K were used to determine the low detection limit of NGHTS. Furthermore, the mixtures of different plasmid DNAs of *C. trachomatis* genotype of F/G, E/J and E/F were prepared at the ratio of 50/50, 30/70, 20/80, 10/90, 2.5/97.5, 1/99, and amplified and sequenced to assess the sensitivity and accuracy of NGHTS in distinguishing mixed *C. trachomatis* genotypes.

### Amplification of minor genotypes using genotype-specific primers in the samples infected with mixed *Chlamydia trachomatis* genotypes

The genotype-specific primers for minor genotypes were designed using DNASTAR software (DNASTAR Inc., Madison, WI, United States) according to the sequence difference between *C. trachomatis* genotypes (Supplementary Table S3). For the verification of the samples with mixed *C. trachomatis* genotypes, *C. trachomatis om*p*A* fragment VS1–VS2 was first amplified using the outer primes *ompA* CT-HTS-F-outer/CT-HTS-R-outer followed by the amplification using the sample-genotype-specific primers. For each sample with mixed-genotype infections, the genotype-specific primer was designed to match the sequence of minor genotype but not the major genotype, especially in the 3′ end. The PCR products were detected by electrophoresis on 2% agarose gel and sent to Ruibo Bioteh (Guangzhou, China) for Sanger sequencing. Genotype verification was conducted by using the BLAST program as previously described (Yang et al., 2010).

### Detection of bacterial load of *Chlamydia trachomatis*

A real-time quantitative PCR (qPCR) assay was adapted to determine *C. trachomatis* bacterial loads using primers of the *Pgp2* gene (Supplementary Table S2). qPCR was carried out in 20 μl reaction volume with 10 μl of TB Green Fast qPCR Mix (Takara Bio Inc., Shiga, Japan), 0.8 μl of forward and reverse primers (10 pmol/ul), 6.4 μl of H_2_O and 2 μl DNA template. The following are the reaction conditions with LightCycler 480 System (Roche Diagnostics GmbH, Mannheim, Germany): 95°C for 30 s, followed by 40 cycles of 95°C for 30 s, 57°C for 30 s and 72°C for 30 s. The bacterial loads were calculated according to the standard curve.

### Statistical analysis

Statistical analysis was done using SPSS 25.0 software (IBM). Continuous variables were presented as mean ± SE and tested by *t*-test whereas categorical variables were expressed as numbers and tested by Chi-square tests. Association of *C. trachomatis* genotype or mixed-genotype with clinical manifestations was explored by multivariate logistic regression analysis and presented as odds ratio (*OR*).

Propensity scores (PS) was calculated using logistic regression with respect to age, clinical departments, antibiotic usage, HPV infection. In addition, PS was adjusted by a standardized mortality ratio weighting (SMRW) method in which a weight of 1 was assigned for cases and a weight of [PS (1 − Pt)]/[(1 − PS) Pt] for controls, respectively. The proportion of treatment (Pt) was calculated by the number of cases / the number of cases plus controls. Different clinical manifestations between *C. trachomatis* genotypes were then compared using the PS-adjusted pseudo-population created by the statistical procedures and presented as adjusted odds ratio (a*OR*).

## Results

### Performance of NGHTS for identifying mixed genotypes

**Figure 1:**
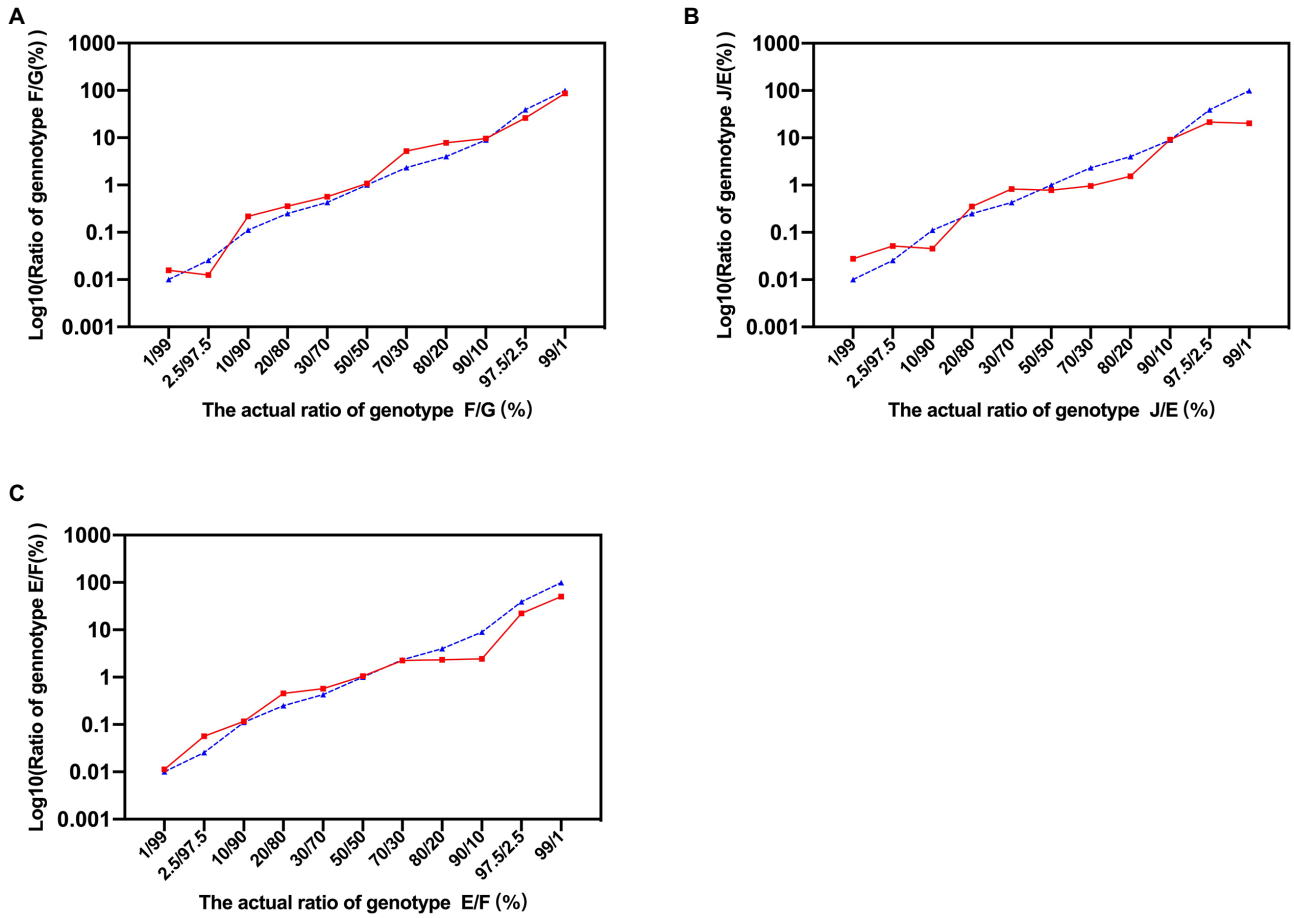
Evaluation of next generation high-throughput sequencing (NGHTS) to quantify the composition of different *Chlamydia trachomatis* genotypes. Plasmid DNAs of different *C. trachomatis* genotypes F/G **(A)**, J/E **(B)**, E/F **(C)** were mixed at the ratio of 1–99, and were amplified and sequenced. The blue dash line and solid red line represent the ratios of different *C. trachomatis* genotypes determined by researchers or detected by NGHTS, respectively. The above experiments were repeated twice and the mean values were calculated and presented.

The schematic diagram of the identification of *C. trachomatis* mixed-genotype infection using NGHTS was shown in Supplementary Figure S2. The nested-PCR to construct NGHTS library was capable of amplifying 10 copies per reaction of the recombinant plasmid DNAs for 8 *C. trachomatis* genotypes (Supplementary Figure S3). To further assess the sensitivity and accuracy of NGHTS in distinguishing mixed *C. trachomatis* genotypes, we prepared a series of plasmid DNA mixtures of two *C. trachomatis* genotypes at the ratio of 1–99, including genotype F and G, J and E as well as E and F. The proportion of different genotypes determined by NGHTS was excellently correlated with the ratio we prepared (Figure 1). For example, when we added 1% of the minor genotype into the mixture, the proportion of the minor genotype determined by NGHTS ranged from 1.11 to 4.69%, suggesting that NGHTS could detect at least 1% of the minor genotype in clinical samples with mixed-genotype infection.

Next, we designed genotype-specific primers to amplify the minor genotypes identified by NGHTS. We found that these genotype-specific primers could specifically amplify the minor genotypes when using plasmid DNAs as templates (Supplementary Figure S4A) and in the samples with mixed genotypes of *C. trachomatis* (Supplementary Figure S4B). We also used PCR products of 7 samples infected with two genotypes to transfect *E. coli* cells. Ten colonies per sample were randomly picked up and sequenced to determine their genotypes of *C. trachomatis*. We found that 5 out of the 7 samples contained the same *C. trachomatis* genotypes as those determined by NGHTS although the proportion of the genotypes were slightly different from the data obtained by NGHTS (Supplementary Table S4). Taken together, our results confirmed the good performance of NGHTS in identifying the composition and proportion of *C. trachomatis* genotypes in the clinical samples.

### Prevalence and genotype distribution of *Chlamydia trachomatis* infection

**Figure 2:**
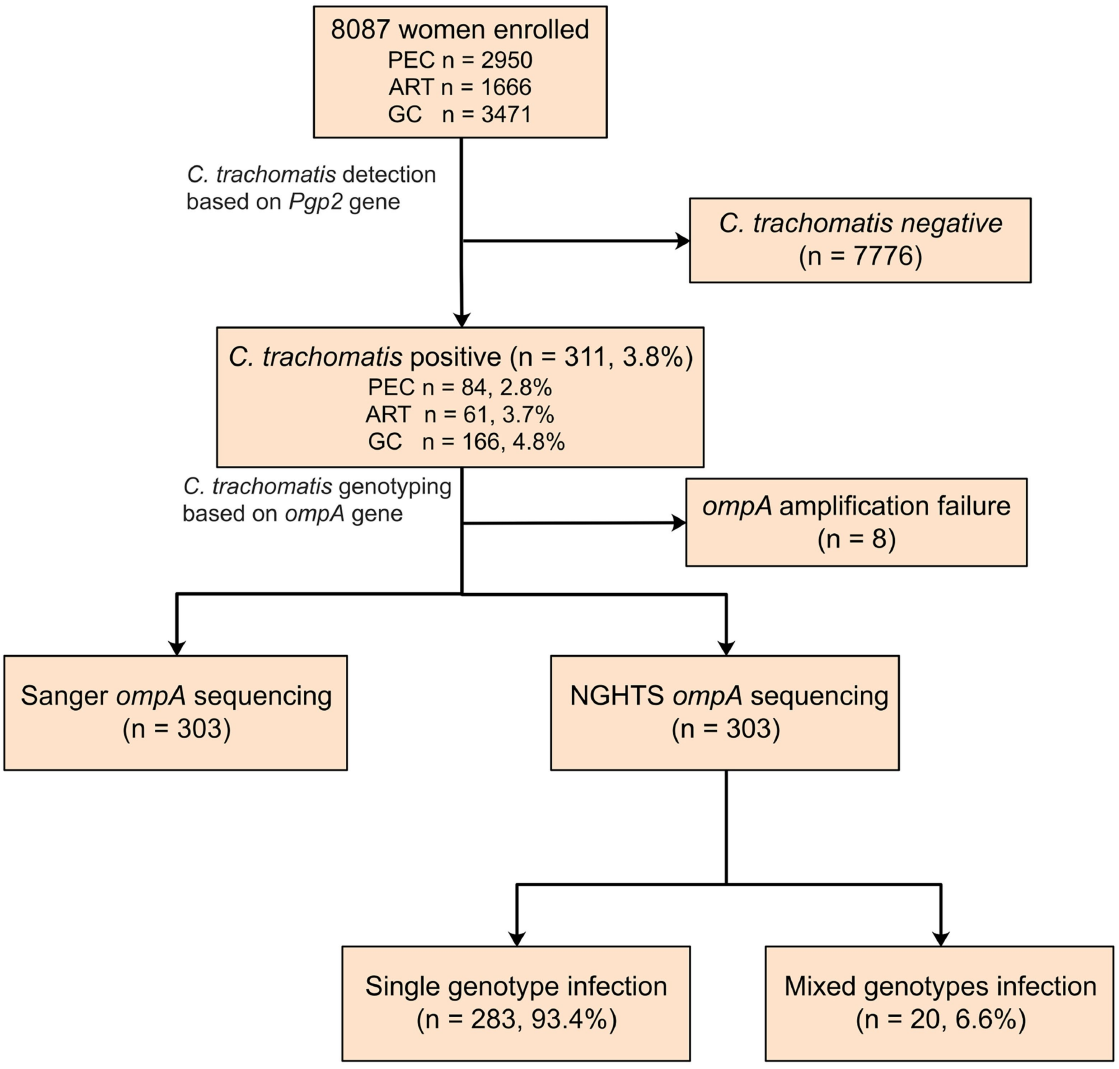
Flow chart for detection and genotyping of *C. trachomatis* in an observational study. The subjects were enrolled from physical examination center (PEC), assisted reproductive technology center (ART), or gynecology clinics (GC), respectively in a hospital in Chenzhou, China. They were first screened for *C. trachomatis* nucleic acid by using polymerase chain reaction (PCR) targeting *Pgp2* gene followed by Sanger sequencing or NGHTS to determine genotypes.

A total of 8,087 participants were tested for *C. trachomatis Pgp2* gene and 311 (3.8, 95% confidence interval [CI] 3.4–4.2%) were found to be positive (Figure 2). The prevalence of *C. trachomatis* infection was 2.8, 3.7 and 4.8% for the participants from PEC, ART and GC, respectively (Figure 2). The *ompA* gene was successfully amplified and sequenced in 97.4% (303/311) *C. trachomatis* pgp2-positive samples, and was classified into 8 *C. trachomatis* genotypes and 3 genogroups proposed by Yuan et al. (1989). The most common three *C. trachomatis* genotypes were E (27.4%, 83/303), F (21.5%, 65/303) and J (18.2%, 55/303, Table 1). Two subjects (0.66%, 2/303) were infected with genotype B (Table 1). For the subjects infected with single *C. trachomatis* genotype, the genotyping results of NGHTS and Sanger sequencing were identical (Supplementary Table S5). For the 8 *C. trachomatis pgp2*-positive samples without genotyping results, both Sanger sequencing primers and NGHTS primers failed to amplify the target *ompA* gene.

**Table 1:** Distribution of *C. trachomatis* genotypes in 303 *C. trachomatis* positive subjects according to *ompA* sequencing results in Chenzhou, China during 2019 and 2021.

Genogroup	Genotype	No. (%) (*N* = 303)
B complex	B	2 (0.66)
	D	40 (13.20)
	E	83 (27.39)
F, G group	F	65 (21.45)
	G	18 (5.94)
C-complex	H	10 (3.30)
	J	55 (18.15)
	K	10 (3.30)
Mixed		20 (6.60)
Total		303 (100.0)

Moreover, NGHTS identified 6.6% (20/303) *C. trachomatis* positive samples to be infected with two (*n* = 18) or three (*n* = 2) *C. trachomatis* genotypes (Table 2). The most frequent two genotypes observed in the mixed-genotype infections were F (55.0%, 11/20) and E (50.0%, 10/20), respectively. Co-infection of genotype F and G accounted for 20.0% (4/20) of the samples with mixed genotypes (Table 2). Of note, among the 20 samples infected with mixed *C. trachomatis* genotypes, 10 (50%) samples were dominated by one *C. trachomatis* genotype, i.e., the proportion of the major genotype >90% (Table 2). In addition, we measured the bacterial load of *C. trachomatis* using qPCR in 17 samples with mixed-genotype infections and 232 samples of single-genotype infection. Although the bacterial load was slightly higher in the mixed-genotype infections than in the single-genotype infections, i.e., 1.36 × 10^5^ (IQR: 6.00 × 10^4^, 4.37 × 10^5^) copies/mL vs. 0.85 × 10^5^ (IQR: 1.44 × 10^4^, 3.97 × 10^5^) copies/mL, the difference was not statistically significant (*p* = 0.476).

**Table 2:** Composition of *C. trachomatis* genotypes in 20 specimens of mixed-genotype infections using high-throughput sequencing in Chenzhou, China during 2019 and 2021.

Ratio of major versus minor genotype	Sample ID	Total sequencing reads	*C. trachomatis* genotypes (reads, %)	
			Major genotype	Minor genotype
≤9	CZ-13154	2865633	**D** 1495589**(52.19)**	**G** 1369971**(47.81)**
	CZ-2906	107674	**D** 57498**(53.40)**	**E** 50172**(46.60)**
	CZ-7399	2114109	**F** 1267351**(59.95)**	**H** 838830**(39.68)**
	CZ-3706	2080328	**J** 1266951**(60.90)**	**E** 804196**(38.66)**
	CZ-8033	1630749	**D** 1023163**(62.74)**	**F** 599935**(36.79)**
	CZ-9288	130285	**D** 90742**(69.65)**	**F** 39218**(30.10)**
	CZ-3612	1695345	**F** 1235996**(72.91)**	**E** 458921**(27.07)**
	CZ-2117	1088398	**E** 882877**(81.12)**	**H** 185179**(17.01)**
				**K** 14015**(1.29)**
	CZ-2021	191006	**E** 161181**(84.39)**	**J** 26375**(13.81)**
				**F** 3116**(1.63)**
	CZ-4061	2242349	**J** 1996995**(89.06)**	**F** 245339**(10.94)**
>9	CZ-1761	124818	**D** 116786**(93.57)**	E 8027**(6.43)**
	CZ-29060	79424	**G** 77444**(97.51)**	**F** 1244**(1.57)**
	CZ-1708	1895050	**H** 1848791**(97.56)**	**E** 39242**(2.07)**
	CZ-10505	1729932	**E** 1690609**(97.73)**	**J** 26740**(1.55)**
	CZ-1860	145204	**J** 142371**(98.05)**	**E** 2829**(1.95)**
	CZ-23209	197318	**F** 193812**(98.22)**	**G** 3480**(1.76)**
	CZ-2477	139817	**G** 137418**(98.28)**	**F** 1486**(1.06)**
	CZ-29117	99617	**K** 98083**(98.46)**	**J** 1521**(1.53)**
	CZ-28087	278981	**G** 275295**(98.68)**	**F** 3574**(1.28)**
	CZ-15863	61654	**E** 60889**(98.76)**	**F** 757**(1.23)**

### Association between clinical manifestations and *Chlamydia trachomatis* genotypes

For the 303 genotype-defined subjects, no significant difference was observed in terms of age, clinical departments, antibiotic usage and HPV infection (*p* > 0.2, Supplementary Table S6). Similar results were obtained between the subjects with single or mixed-genotype infections (*p* > 0.3, Supplementary Table S6). In our study, 70.1% (218/303) of the participants reported 14 symptoms or signs including vaginosis (38.3%), PID (10.9%) and cervicitis (8.4%; Supplementary Table S7). Of note, only 29.9% (93/311) of subjects were asymptomatic *C. trachomatis* infection. However, the percentage of asymptomatic *C. trachomatis* infection was significantly higher (71.3%) in PEC compared to ART (23.7%) and GC (10.4%, *p* < 0.001, Table 3). Co-infection with HPV increased the risk of symptomatic *C. trachomatis* infection (83.1% vs. 67.2%, *p* = 0.01) and cervical squamous intraepithelial lesion (23.7% vs. 8.5%, *p* < 0.001, Table 3).

**Table 3:** Clinical manifestations of *C. trachomatis*-infected women with respect to age, clinical departments, antibiotic usage and HPV infection.

Manifestations	Age (year)				*p*^a^ value	Clinical departments^b^			*p* value	Antibiotic usage (previous 3 months)		*p* value	HPV infection		*p* value
	≤25 *n* = 63	25–35 *n* = 140	35–45 *n* = 58	>45 *n* = 42		PEC *n* = 80	ART *n* = 59	GC *n* = 164		Yes *n* = 14	No *n* = 289		Yes *n* = 71	No *n* = 232	
Asymptomatic (%)															
Yes	20.6	27.9	32.8	40.5	0.15	71.3	23.7	10.4	**<0.001**	7.1	30.1	0.122	16.9	32.8	**0.01**
No	79.4	72.1	67.2	59.5		28.8	76.3	89.6		92.9	69.9		83.1	67.2	
Vaginosis (%)															
Yes	52.4	39.3	29.3	28.6	**0.03**	16.3	32.2	51.8	**<0.001**	57.1	37.7	0.145	45.1	36.6	0.202
No	47.6	60.7	70.7	71.4		83.8	67.8	48.2		42.9	62.3		54.9	63.4	
Pelvic inflammatory disease (%)															
Yes	6.3	14.3	17.2	0.0	**0.017**	0.0	33.9	8.5	**<0.001**	42.9	9.7	**0.001**	8.5	12.1	0.398
No	93.7	85.7	82.8	100.0		100.0	66.1	91.5		57.1	90.3		91.5	87.9	
Cervicitis (%)															
Yes	6.3	7.9	10.3	11.9	0.726	12.5	10.2	6.1	0.218	0.0	9.0	0.493	8.5	8.6	0.964
No	93.7	92.1	89.7	88.1		87.5	89.8	93.9		100.0	91.0		91.5	91.4	
Vagina cleanliness (%)															
I/II	31.6	35.3	41.7	40.6	0.97	46.8	44.8	18.3	**<0.001**	41.7	36.2	0.671	34.6	37.2	0.439
III	36.8	37.3	33.3	31.3		40.5	34.5	31.0		41.7	35.2		30.8	37.2	
IV	31.6	27.5	25.0	28.1		12.7	20.7	50.7		16.7	28.6		34.6	25.6	
Cervical abnormalities (%)^c^															
Benign	76.6	82.8	83.3	86.1	0.792	88.6	83.9	78.1	0.371	87.5	82.0	0.78	62.7	88.3	**<0.001**
ASC-US	10.6	5.2	4.2	2.8		2.5	6.5	7.3		0.0	5.9		13.6	3.2	
SIL	12.8	12.1	12.5	11.1		8.9	9.7	14.6		12.5	12.1		23.7	8.5	

^a^*p* values were calculated using Chi-square tests. Bold *p* value indicate statistically significant, i.e., *p* < 0.05.^b^PEC, physical examination center; ART, assisted reproductive technology; GC, gynecology clinics.^c^ASC-US, atypical squamous cells of undetermined significance; SIL, squamous intraepithelial lesion.

In addition, patients from GC were more likely to present with vaginosis (51.8%, *p* < 0.001) and vaginal cleanliness of degree IV (50.7%, *p* < 0.001) compared to the subjects from PEC (16.3% for vaginosis and 12.7% for degree IV) and ART (32.2% for vaginosis and 20.7% for degree IV, Table 3). PID was more often recorded in the patients from ART (33.9%) compared to the subjects from PEC (0%) or GC (8.5%, *p* < 0.001). In our study, PID was also more frequently observed in older subjects (*p* = 0.017) and those with antibiotic usage history (*p* = 0.001). Furthermore, vaginosis was more likely diagnosed in younger women, especially those under 25 years old (*p* = 0.03, Table 3).

The association between *C. trachomatis* genogroup or genotypes and clinical manifestations was analyzed by multivariate logistic regression among 283 subjects infected with single *C. trachomatis* genotype after controlling the parameters of age, clinical departments, antibiotic usage and HPV infection (Table 4). Compared to genotype E, subjects infected with genotype G were more often diagnosed as PID (27.8% vs. 9.6%, OR = 6.06, 95%CI, 1.29–28.5; *p* = 0.023, Table 4) and vagina cleanliness of degree IV (40.0% vs. 27.3%, *OR* = 6.91, 95%*CI*, 1.25–38.1, *p* = 0.026, Table 4). Further analysis of propensity score reweighting data using the SMRW method confirmed the association of *C. trachomatis* genotype G infection with PID (a*OR* = 3.61, 95% *CI*, 1.02–12.8, *p* = 0.046, Supplementary Table S8), but not vagina cleanliness of degree IV (a*OR* = 3.00, 95% *CI*, 0.65–13.9, *p* = 0.161, Supplementary Table S8) even though all other parameters were well balanced after weighting (Supplementary Table S9).

**Table 4:** Relationships of clinical manifestations to *C. trachomatis* genotypes in 283 women infected with single-genotype of *C. trachomatis*.

Manifestations	*C. trachomatis* genotype (*n* = 261)^a^					*C. trachomatis* genogroup (*n* = 283)		
	E *n* = 83	D *n* = 40	F *n* = 65	G *n* = 18	J *n* = 55	B-complex *n* = 125	F/G group *n* = 83	C-complex *n* = 75
Asymptomatic								
Yes (*n*, %)	22 (26.5)	15 (37.5)	20 (30.8)	6 (33.3)	13 (23.6)	37 (29.6)	26 (31.3)	20 (26.7)
*OR* (95% *CI*)^b^	Ref^c^	1.58 (0.56–4.51)	0.81 (0.33–1.99)	0.69 (0.18–2.67)	0.78 (0.3–2.05)	Ref	0.7 (0.33–1.51)	0.91 (0.42–2.0)
Vaginosis								
Yes (*n*, %)	33 (39.8)	14 (35.0)	20 (30.8)	5 (27.8)	28 (50.9)	48 (38.4)	25 (30.1)	35 (46.7)
*OR* (95% *CI*)	Ref	0.87 (0.37–2.06)	0.79 (0.38–1.64)	0.73 (0.22–2.42)	1.75 (0.84–3.66)	Ref	0.8 (0.43–1.51)	1.43 (0.77–2.66)
Pelvic inflammatory disease								
Yes (*n*, %)	8 (9.6)	2 (5.0)	7 (10.8)	5 (27.8)	9 (16.4)	10 (8.0)	12 (14.5)	10 (13.3)
OR (95% *CI*)	Ref	0.32 (0.04–2.41)	1.44 (0.42–4.9)	**6.06 (1.29–28.5)**	1.54 (0.48–4.91)	Ref	2.71 (0.95–7.78)	1.49 (0.52–4.3)
Cervicitis								
Yes (*n*, %)	5 (6.0)	4 (10.0)	8 (12.3)	2 (11.1)	3 (5.5)	9 (7.2)	10 (12.0)	5 (6.7)
*OR* (95% *CI*)	Ref	1.71 (0.41–7.05)	2.07 (0.63–6.86)	1.88 (0.32–10.9)	0.89 (0.2–3.94)	Ref	1.62 (0.61–4.32)	0.92 (0.29–2.94)
Vagina cleanliness (vs. I/II)								
III (*n*, %)	16 (29.1)	9 (36.0)	17 (37.8)	6 (40.0)	18 (45.0)	26 (31.7)	23 (38.3)	21 (39.6)
*OR* (95% *CI*)	Ref	1.71 (0.52–5.58)	1.43 (0.56–3.67)	3.16 (0.65–15.32)	2.39 (0.88–6.49)	Ref	1.39 (0.62–3.14)	1.48 (0.64–3.45)
IV (*n*, %)	15 (27.3)	7 (28.0)	9 (20.0)	6 (40.0)	10 (25.0)	22 (26.8)	15 (25.0)	14 (26.4)
*OR* (95% *CI*)	Ref	2.49 (0.6–10.29)	0.89 (0.28–2.87)	**6.91 (1.25–38.09)**	1.59 (0.49–5.12)	Ref	1.29 (0.49–3.38)	1.18 (0.44–3.15)
Cervical abnormalities (vs. Benign)^d^								
ASC-US (*n*, %)	4 (6.0)	2 (5.7)	1 (1.8)	0	3 (7.7)	6 (5.8)	1 (1.4)	6 (10.7)
*OR* (95% *CI*)	Ref	0.71 (0.11–4.77)	0.32 (0.03–3.42)	NA^e^	0.84 (0.15–4.73)	Ref	0.31 (0.04–2.81)	1.79 (0.5–6.48)
SIL (*n*, %)	4 (6.0)	6 (17.1)	9 (16.4)	1 (6.7)	4 (10.3)	10 (9.6)	10 (14.3)	6 (10.7)
*OR* (95% *CI*)	Ref	2.96 (0.71–12.34)	3.44 (0.94–12.58)	1.15 (0.11–11.98)	1.67 (0.38–7.41)	Ref	1.82 (0.68–4.88)	1.21 (0.4–3.69)

^a^Genotypes of < 5% of the total patients were analyzed only in their corresponding genogroups.^b^Odds ratio (*OR*) was calculated in multivariate logistic regression analysis in which the parameters of age, clinical departments, antibiotic usage and HPV infection were under control. Bold *OR* and a*OR* indicate statistically significant, i.e., *p* < 0.05.^c^Genotype E and B-complex genogroup were set as reference group, respectively.^d^ASC-US, atypical squamous cells of undetermined significance; SIL, squamous intraepithelial lesion.^e^NA, not applicable.

### Comparison of clinical manifestations between *Chlamydia trachomatis* single and mixed-genotype infections

Although the subjects with *C. trachomatis* single and mixed-genotype infections reported similar symptoms, mixed-genotype infections were more likely to result in worse vagina cleanliness of degree IV (*OR* = 8.61, 95%*CI*, 1.53–48.5; *p* = 0.015, Table 5) than single genotype infections. Further stratified analysis revealed that the occurrence of worse vagina cleanliness and cervical SIL (*OR* = 5.76, 95%*CI*, 1.06–31.20, *p* = 0.042) was mainly observed in the mixed genotype-infected subjects whose minor *C. trachomatis* genotype was ≥10% (Table 5).

**Table 5:** Comparison of clinical manifestations between women with *C. trachomatis* single-genotype and mixed-genotype infections.

Manifestations	Single genotype event/*N* (%)	*C. trachomatis* mixed-genotype infection^a^								
		Total (*N* = 20)			Without dominant genotype (*N* = 10)			With dominant genotype (*N* = 10)		
		Event/*N* (%)	*OR*^b^ (95% *CI*)	a*OR*^c^ (95% *CI*)	Event/*N* (%)	OR (95% CI)	a*OR* (95% *CI*)	Event/*N* (%)	*OR* (95% *CI*)	a*OR* (95% *CI*)
Asymptomatic	83/283 (29.3)	5/20 (25.0)	0.83 (0.24–2.86)	0.80 (0.28–2.28)	1/10 (10.0)	0.67 (0.08–5.64)	0.41 (0.05–3.29)	4/10 (40.0)	0.89 (0.19–4.15)	0.86 (0.24–3.12)
Vaginosis	108/283 (38.2)	9/20 (45.0)	1.52 (0.57–4.03)	1.33 (0.54–3.32)	6/10 (60.0)	1.91 (0.51–7.16)	2.22 (0.61–8.03)	3/10 (30.0)	1.19 (0.27–5.24)	1.21 (0.3–4.78)
Pelvic inflammatory disease	32/283 (11.3)	2/20 (10.0)	0.6 (0.11–3.21)	0.88 (0.20–3.98)	0	NA	NA	2/10 (20.0)	1.3 (0.18–9.19)	1.43 (0.29–6.96)
Cervicitis	24/283 (8.5)	2/20 (10.0)	1.1 (0.23–5.16)	1.20 (0.26–5.49)	1/10 (10.0)	1.5 (0.17–12.92)	1.68 (0.2–14.0)	1/10 (10.0)	0.93 (0.11–8.13)	0.71 (0.09–5.79)
Vagina cleanliness (vs. I/II)										
III	70/195 (35.9)	4/13 (30.8)	2.46 (0.43–14.24)	2.11 (0.37–11.87)	0	NA	NA	4/8 (50.0)	2.69 (0.46–15.78)	2.07 (0.37–11.64)
IV	51/195 (26.2)	7/13 (53.8)	**8.61 (1.53–48.53)**	**5.17 (1.03–25.92)**	5/5 (100.0)	NA ^d^	NA	2/8 (25.0)	3.44 (0.43–27.73)	1.41 (0.19–10.35)
Cervical abnormalities (vs. Benign)^e^										
ASC-US	13/230 (5.7)	1/17 (5.9)	1.18 (0.12–11.35)	1.22 (0.15–10.12)	0	NA	NA	1/10 (10.0)	2.14 (0.2–23.5)	1.85 (0.21–15.91)
SIL	26/230 (11.3)	4/17 (23.5)	2.8 (0.75–10.5)	2.44 (0.73–8.13)	3 (42.9)	**5.76 (1.06–31.22)**	**5.51 (1.17–26.01)**	1/10 (10.0)	1.06 (0.11–10.25)	0.92 (0.11–7.64)

^a^Mixed-genotype infections were divided into two groups with or without dominant genotype in which the proportion of minor genotypes was <10% or ≥10%, respectively.^b^Odds ratio (*OR*) was calculated in multivariate logistic regression analysis in which the parameters of age, clinical departments, antibiotic usage and HPV infection were under control. ^c^adjusted odd ratio (a*OR*) was calculated based on the data of propensity score weighting. Bold *OR* and a*OR* indicate statistically significant, i.e., *p* < 0.05.^d^NA, not applicable.^e^ASC-US, atypical squamous cells of undetermined significance; SIL, squamous intraepithelial lesion.

We then conducted propensity score reweighting analysis to balance the factors of age, clinical departments, antibiotic usage, HPV infection and major *C. trachomatis* genotypes (Supplementary Table S10). Further logistic regression analysis confirmed the association of worse vagina cleanliness (a*OR* = 5.17, 95%*CI*, 1.03–25.9, *p* = 0.046) and cervical SIL (a*OR* = 5.51, 95%*CI*, 1.17–26.01, *p* = 0.031) with mixed-genotype infection when compared to single-genotype infection in particular for the mixed-genotype infections in which minor *C. trachomatis* genotype was ≥10% (Table 5). These results indicated that mixed-genotype infections of *C. trachomatis* may be associated with worse vaginal inflammation and cervical squamous intraepithelial lesion.

## Discussion

In this study, a prevalence of 3.8% of current *C. trachomatis* infection was documented in the subjects who visited Chenzhou Hospital of China for either annual physical examination, diagnosis or treatment of infertility or gynecological diseases. The predominant *C. trachomatis* genotypes were E, F and J, which are similar to our previous findings (Chen et al., 2020). In addition, genotype B, an ocular genotype to cause ocular infection, was detected in two subjects in our study. However, genotype B ocular strain may be a recombinant strain with a urogenital genomic backbone and ocular genotype B *ompA* insert. Similar findings have been reported in one subject in Zheng et al. study and four subjects in Lesiak-Markowicz et al. study, respectively (Zheng et al., 2007; Lesiak-Markowicz et al., 2019).

In our study, we adapted NGHTS technology to determine *C. trachomatis* genotypes in particular mixed-genotype infections. Our results indicated that both NGHTS and Sanger sequencing correctly identified *C. trachomatis* genotypes in 283 subjects who infected with single genotype of *C. trachomatis.* Moreover, NGHTS was able to identify the presence and proportion of mixed C. *trachomatis* genotypes in 6.6% of *C. trachomatis* positive samples. We further found that mixed-genotype infections were associated with worse vaginal cleanliness and cervical SIL. To the best of our knowledge, this is the first study to assess the feasibility of NGHTS to identify and quantify mixed-genotype infection of *C. trachomatis* in China. Our preliminary results support NGHTS as a simple and useful method for differentiating *C. trachomatis* genotypes and determining mixed-genotype infections.

There are several genotyping methods for *C. trachomatis* including high-resolution multilocus sequence typing (hr-MLST) of multiple genes, and one serovar may consist of several different sequence types (STs) of *C. trachom*atis (Versteeg et al., 2015). However, sequencing of the *ompA* gene is still widely used to determine the serovars or genotypes of *C. trachomatis* and the results are highly associated with the serotyping methods using a large panel of monoclonal antibodies (Mukherjee et al., 2011). We would like to emphasize that the purpose of our study is to identify mixed infections of *C. trachomatis* and their pathological impact, not to accurately determine the sequence types of *C. trachomatis* in the clinical samples.

Sanger sequencing is a powerful tool for genotyping and has been used to determine *C. trachomatis* genotypes. However, it may not be suitable for identifying mixed-genotype infections of *C. trachomatis* since Sanger sequencing only produces one consensus sequence according to the alignment results of multiple sequencing data and the intensity of sequencing signals at each point; hence, only the dominant genotype sequence could be identified (Gharsallah et al., 2018). Although the mixed-genotype infections can be identified by PCR-RFLP, hybridization methods and DNA microarray assay, these assays are not sensitive enough to identify all the mixed-genotype infections and cannot quantify genotype proportions in mixed-genotype infections. In addition, these assays cannot detect novel mutations or genovariants (Gharsallah et al., 2012). NGHTS has opened a new frontier to characterize the composition of complex populations of microbes and to uncover novel sequences or mutations, and it overcomes the constraints of Sanger sequencing and can achieve greater than 10,000 base pair coverage per sample (Shendure and Ji, 2008). Meanwhile, NGHTS is capable of obtaining tens or even hundreds of thousands of reads simultaneously, and the sensitivity of detecting low-frequency mutation sites and minor sequences has been dramatically enhanced. For example, Quer et al. (2015) have reported that NGHTS is a reliable method for genotyping hepatitis C virus (HCV) and identifying mixed-genotype infections of HCV through a phylogenetic classification of sequencing reads of HCV *NS5B* gene. In our study, we found that NGHTS could readily determine different proportions of *C. trachomatis* genotypes D-K at the ratio of 1:1.1–1:92.7 in clinical samples. In contrast, the detection limit of microarray assay was about 1:5, which lags far behind the detection performance of NGHTS. Although NGHTS methodology is not simple and needs support of research laboratories, we have assessed the possibility of NGHTS in large-scale implementation by labeling primers with different barcode sequences and mixing 10 samples in one NGHTS reaction. Therefore, NGHTS will dramatically improve the efficiency and greatly reduce the cost for identifying *C. trachomatis* genotypes. Additionally, the potential quantitation bias caused by nested-PCR should be taken into account, thus the NGHTS is only a relatively quantitative method.

It has been reported that the prevalence of *C. trachomatis* mixed-genotype infection varied from 2 to 19% (Stothard, 2001; Molano et al., 2004; Xiong et al., 2006; Zhang et al., 2012). It remains to elucidate if mixed-genotype infections represent co-infection or superinfection of different *C. trachomatis* genotypes. Hsu et al. (2006) proposed that two separate episodes of *C. trachomatis* infection and the lack of immunological cross protection between *C. trachomatis* genotypes may result in mixed-genotype infections. In our study, *C. trachomatis* genotype E and F were more frequently detected in the mixed-genotype infections (Table 2), which may be due to the extensive distribution of these two genotypes in China. Gallo Vaulet and Gharsallah, et al. also reported that 86.7 and 76.9% of the mixed-genotype infections contained genotype E, respectively (Gallo Vaulet et al., 2016; Gharsallah et al., 2018). Interestingly, we found that genotype G only accounted for 5.94% of the total *C. trachomatis* infections in our study population, but was detected in 25.0% of the mixed-genotype infections. It is unclear if genotype G has weaker immunological cross protection than other *C. trachomatis* genotypes. Furthermore, genotype D was more often detected in the mixed-genotype infections particularly when the proportion of minor *C. trachomatis* genotype was ≥10%. Our results might suggest increased susceptibility of genotype D and G in the co- or super-infection of *C. trachomatis.*

Another important issue is the association between specific clinical manifestations and *C. trachomatis* genotypes or mixed-genotype infections. It has been reported that several *C. trachomatis* genotypes such as F, G and K may result in more severe clinical manifestations (Geisler et al., 2003; Molano et al., 2004; Millman et al., 2006; Gao et al., 2007). In the current study, we also observed that *C. trachomatis* genotype G infection was more prone to PID (Table 4). Our results are consistent with the findings of Gao et al. (2007). Till now, there are few studies to compare the difference of clinical manifestations between single and mixed-genotype infections of *C. trachomatis.* Our preliminary results indicated that mixed-genotype infections may result in worse vaginal cleanliness and cervical SIL (Table 5) while we found no significant difference of vaginal cleanliness among different single-genotype *C. trachomatis* infections, suggesting that co- or super-infection and the interaction of different *C. trachomatis* strains may contribute to stronger inflammatory reactions and worse vaginal or cervical damage (Gharsallah et al., 2012). It has been found that cervical SIL and cervical cancer are related to *C. trachomatis* infection (Smith et al., 2004; Castellsagué et al., 2014) or *C. trachomatis*/HPV co-infection (de Paula et al., 2007; da Silva Barros et al., 2012; de Abreu et al., 2012; Magalhães et al., 2015). Our further analysis indicated that *C. trachomatis* mixed-genotype infection was associated with cervical SIL compared with single-genotype infection even controlling for HPV infection (Table 5). Furthermore, Madeleine et al. (2007) reported that *C. trachomatis* genotypes B, D, E, G, I, and J were associated with squamous cell cancer although this association was not supported by other studies (Smith et al., 2004). We also did not observe the association of different *C. trachomatis* genotypes with SIL (Table 4). Previous studies indicated that *C. trachomatis* could inhibit apoptosis of *C. trachomatis*-infected cells, trigger host DNA damage, and induce cell proliferation (Chumduri et al., 2013). These results support *C. trachomatis* infection as a potential cause of SIL (Kun et al., 2013). Therefore, it is reasonable to speculate that mixed-genotype infection may facilitate *C. trachomatis*-associated effects on the development of cervical SIL due to the synergistic effect of multiple genotype infections. Similar synergistic impact has been observed in HPV mixed-genotype infections in which cervical SIL was significantly more frequent in mixed-genotype HPV infection than in single genotype HPV infection (Bachtiary et al., 2002; Bruno et al., 2020; Oyervides-Muñoz et al., 2020; Kim et al., 2021). Further research did show that tumors caused by mixed-genotype infection of HPV had a higher PD-1 expression in tumor-infiltrating lymphocytes (TILs), which could help cancer cells evade the immune response and progress more quickly (Mendoza et al., 2021). Actually, inflammation *per se* is a risk factor for cell malignant transformation (Greten and Grivennikov, 2019). More severe inflammation caused by mixed-genotype infection of *C. trachomatis* may also play a role in the process of cervical SIL during *C. trachomatis* infection.

In addition, previous studies have revealed high recombination rates among *C. trachomatis* strains, indicating their important role in the evolutionary pathways (Gomes et al., 2007). For example, the recombination event between the MOMP genes of genotype I and H may generate the composite MOMP of Ia variant (Lampe et al., 1993). Clinical strains of *C. trachomatis* have been reported to be resistant to tetracycline (Jones et al., 1990), macrolides (Misyurina et al., 2004), even multiple antibiotics (Somani et al., 2000). In an *in vitro* study (Suchland et al., 2009), a co-infection model was used to successfully generate tetracycline-resistant *C. trachomatis* L_2_ strain from co-infection of tetracycline-resistant *C. suis* R19 and a tetracycline-sensitive L_2_ strain. The *ompA* gene of *C. trachomatis* can be inserted into different genomic backbones through potential gene recombination. However, the recombination of *ompA* may happen most likely between L strain and I/J/K strains or between ocular and urogenital branches or between L2b and D-Da strains (Somboonna et al., 2011; Harris et al., 2012; Matičič et al., 2016; Borges et al., 2021). Hadfield et al. (2017) reported that in their comprehensive global genome dynamic analysis of *C. trachomatis*, only serovar D and J appeared in both monophyletic lineage T1 and T2. In addition, recombination did not disrupt the *ompA* gene of the most prevalent genotype E. Furthermore, the *ompA* gene of *C. trachomatis* is a single copy gene and may have a lower probability of switching compared to the multi-copy genes, e.g., cryptic plasmid (Joseph et al., 2011). Dalevi et al. (2002) found that the major factors influencing the structure of the *C. trachomatis* genomes are nucleotide substitutions and deletions, and the frequency of horizontal gene transfer events was low. Although the recombination of the *ompA* gene was mainly reported in case reports, the mixed-genotype infection identified in our study was just based on the *ompA* typing system and did not address the potential recombination of the *ompA* gene. WGS or hr-MLST needs to be performed to address the mixed genotype identified in our study and to identify any potential recombinants arising as a result of mixed infection. Our preliminary results prove the importance of identifying mixed-genotype infections of *C. trachomatis* by using NGHTS as a screening assay. Furthermore, qPCR is more sensitive than conventional PCR in detecting C. trachomatis infection. It is necessary to use qPCR for simultaneous detection to ensure accuracy when NGHTS is used to detect C. trachomatis mixed-genotype infections in clinical samples.

Our study has some limitations. (1) The cross-sectional study cannot determine the cause-effect of mixed-genotype infection of *C. trachomatis* and cervical damage. Our findings should be explained with caution. (2) The number of *C. trachomatis* positive samples is small due to the low prevalence of *C. trachomatis* infection and mixed-genotype infections of *C. trachomatis* although a total of 8,087 clinical samples were screened in our study. However, the sample size of 20 mixed-genotype infections was relatively large compared with previous studies. We also used the propensity scores weighting analysis to ensure the accuracy and reliability of the results. (3) All the subjects were from one hospital and were consecutively recruited from the selected clinics. The selection of the participants may limit the generalizability of the findings. (4) Conventional PCR detection rather than qPCR was used in our study and may underestimate *C. trachomatis* prevalence. The *ompA* target genotyping did not cover the entire *ompA* gene due to the read length limitation of NGHTS. (5) Antibiotic resistance of *C. trachomatis* is an ongoing concern although Hadfield, et al. reported that the comprehensive global genome dynamics of *C. trachomatis* did not show evidence of circulating genomic resistance in *C. trachomatis* (Hadfield et al., 2017). In our study, we did not investigate antibiotic resistance since the purpose of our study was to genotype *C. trachomatis* in the samples analyzed.

We confirmed NGHTS as a useful tool for sensitive and accurate identification of *C. trachomatis* genotypes in particular mixed-genotype infections. Our results revealed the feasibility of NGHTS in characterizing mixed *C. trachomatis* infections and its clinical value. Mixed infections of *C. trachomatis* may associate with worse vaginal cleanliness and cervical SIL. Therefore, NGHTS should be further assessed and implemented as a routine screening assay for diagnosis and genotyping of *C. trachomatis* infections.

## Data availability statement

The datasets presented in this study can be found in online repositories. The names of the repository/repositories and accession number(s) can be found at: https://www.ncbi.nlm.nih.gov/, PRJNA786082.

## Ethics statement

The studies involving human participants were reviewed and approved by Ethical Committee of Chenzhou No. 1 People’s Hospital. The patients/participants provided their written informed consent to participate in this study.

## Author contributions

JZ and JS: analysis and interpretation of data, conduction of experiment, and drafting of manuscript. LL: acquisition of data and conduction of experiment. CA, HL, and LW: performed experiment. YL: performed the data analysis. HW: revision of manuscript. ST and HC: conception, design, and finalizing manuscript. All authors read and approved the final manuscript.

## Funding

This work was supported by the Bureau of Science and Information Technology of Guangzhou Municipality (grant no. 201704020219), Natural Science Foundation of Hunan Province (grant no. 2021JJ70002) and Bureau of Chenzhou Science and Technology (yfzx201908).

## Conflict of interest

The authors declare that the research was conducted in the absence of any commercial or financial relationships that could be construed as a potential conflict of interest.

## Publisher’s note

All claims expressed in this article are solely those of the authors and do not necessarily represent those of their affiliated organizations, or those of the publisher, the editors and the reviewers. Any product that may be evaluated in this article, or claim that may be made by its manufacturer, is not guaranteed or endorsed by the publisher.

## Supplementary material

SUPPLEMENTARY FIGURE S1 Reference sequences alignment and primers design for next generation high-throughput sequencing (NGHTS) targets.

SUPPLEMENTARY FIGURE S2 The schematic diagram of the identification of *Chlamydia trachomatis* (*C. trachomatis*) mixed-genotype infection using the next generation high-throughput sequencing (NGHTS) process. PCR, polymerase chain reaction; BWA, Burrows-Wheeler transform.

SUPPLEMENTARY FIGURE S3 Low detection limit of high-throughput sequencing primers for different *Chlamydia trachomatis* genotypes.

SUPPLEMENTARY FIGURE S4 Verification of genotype-specific primers for amplifying minor *Chlamydia trachomatis* (*C. trachomatis*) genotypes from mixed-genotype infections of *C. trachomatis*. **(A)** Genotype-specific primers can specifically amplify minor *C. trachomatis* genotypes when using plasmid DNAs of mixed-genotypes of *C. trachomatis* as templates. **(B)** The minor genotypes were specifically amplified from the samples of mixed-genotype infections by using genotype-specific primers. NC, negative control; +, PCR positive; -, PCR negative.
