# Supplementary figures and images for: Identification and characterization of mixed infections of Chlamydia trachomatis via high-throughput sequencing
Source: Front Microbiol. 2022 Nov 10;13:1041789. doi: 10.3389/fmicb.2022.1041789 (PMC9687396; doi:10.3389/fmicb.2022.1041789)

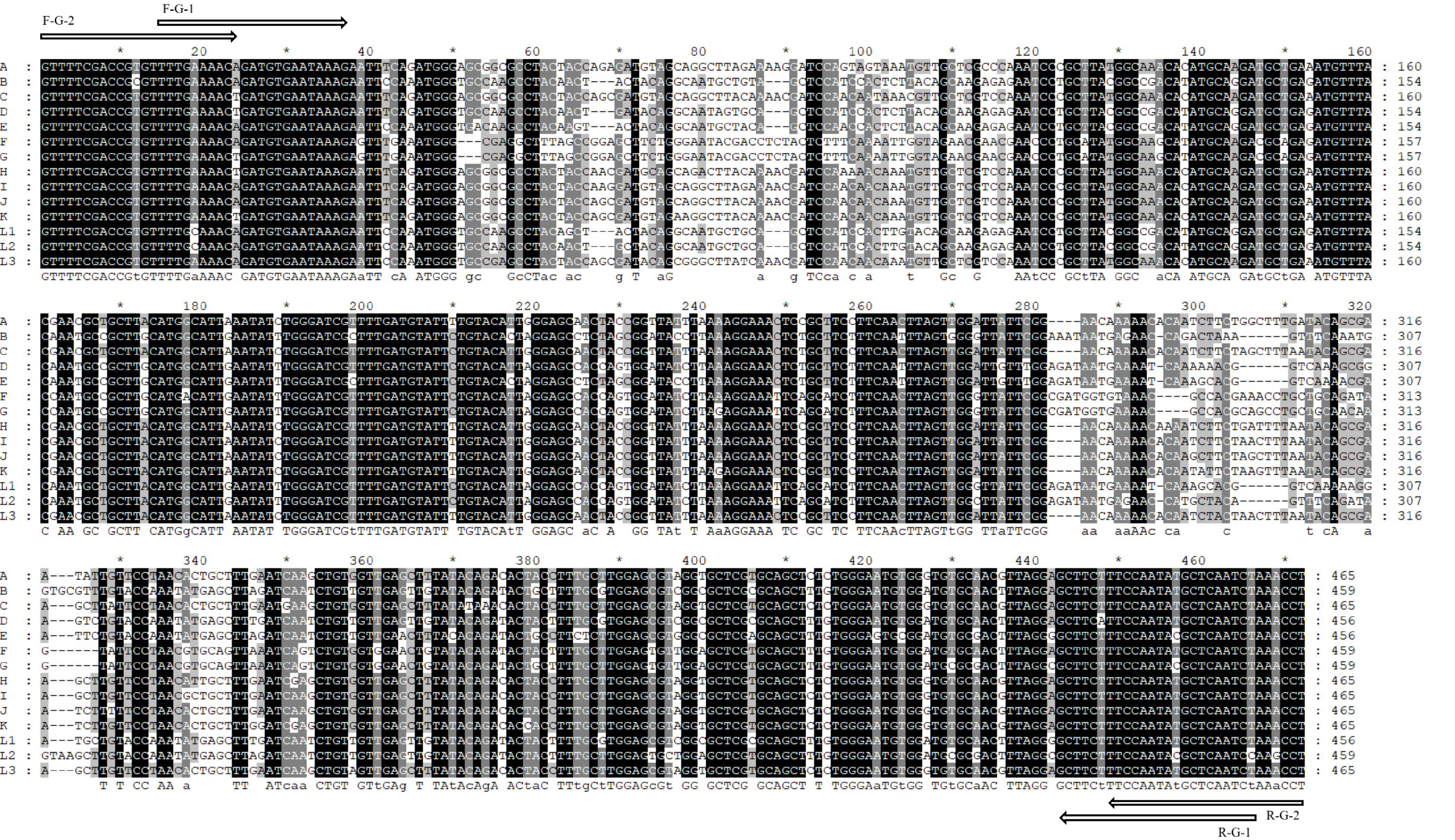

Supplement: SUPPLEMENTARY FIGURE S1 — Reference sequences alignment and primers design for next generation high-throughput sequencing (NGHTS) targets. [file Image_1.JPEG]

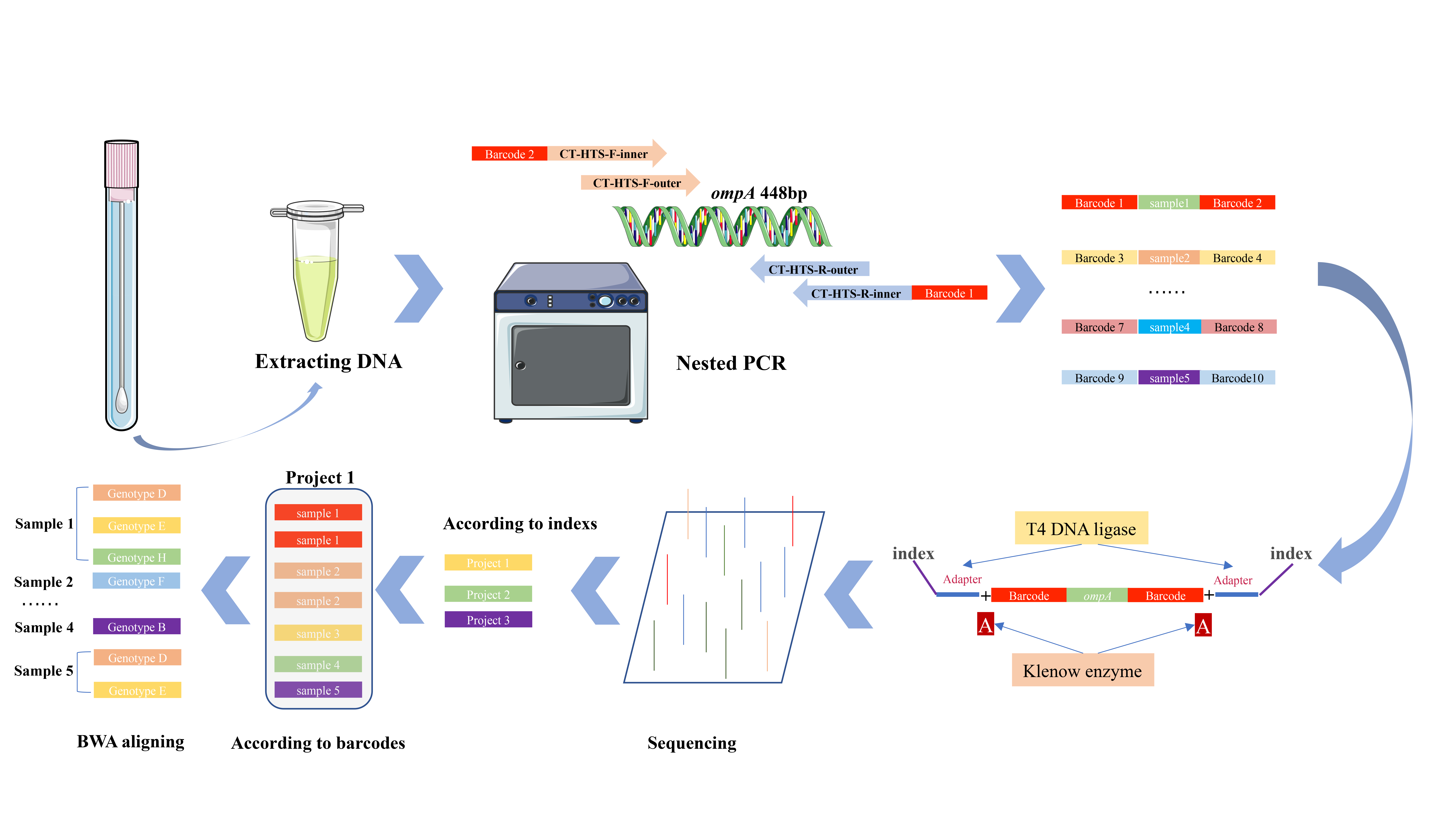

Supplement: SUPPLEMENTARY FIGURE S2 — The schematic diagram of the identification of Chlamydia trachomatis (C. trachomatis) mixed-genotype infection using the next generation high-throughput sequencing (NGHTS) process. PCR, polymerase chain reaction; BWA, Burrows-Wheeler transform. [file Image_2.JPEG]

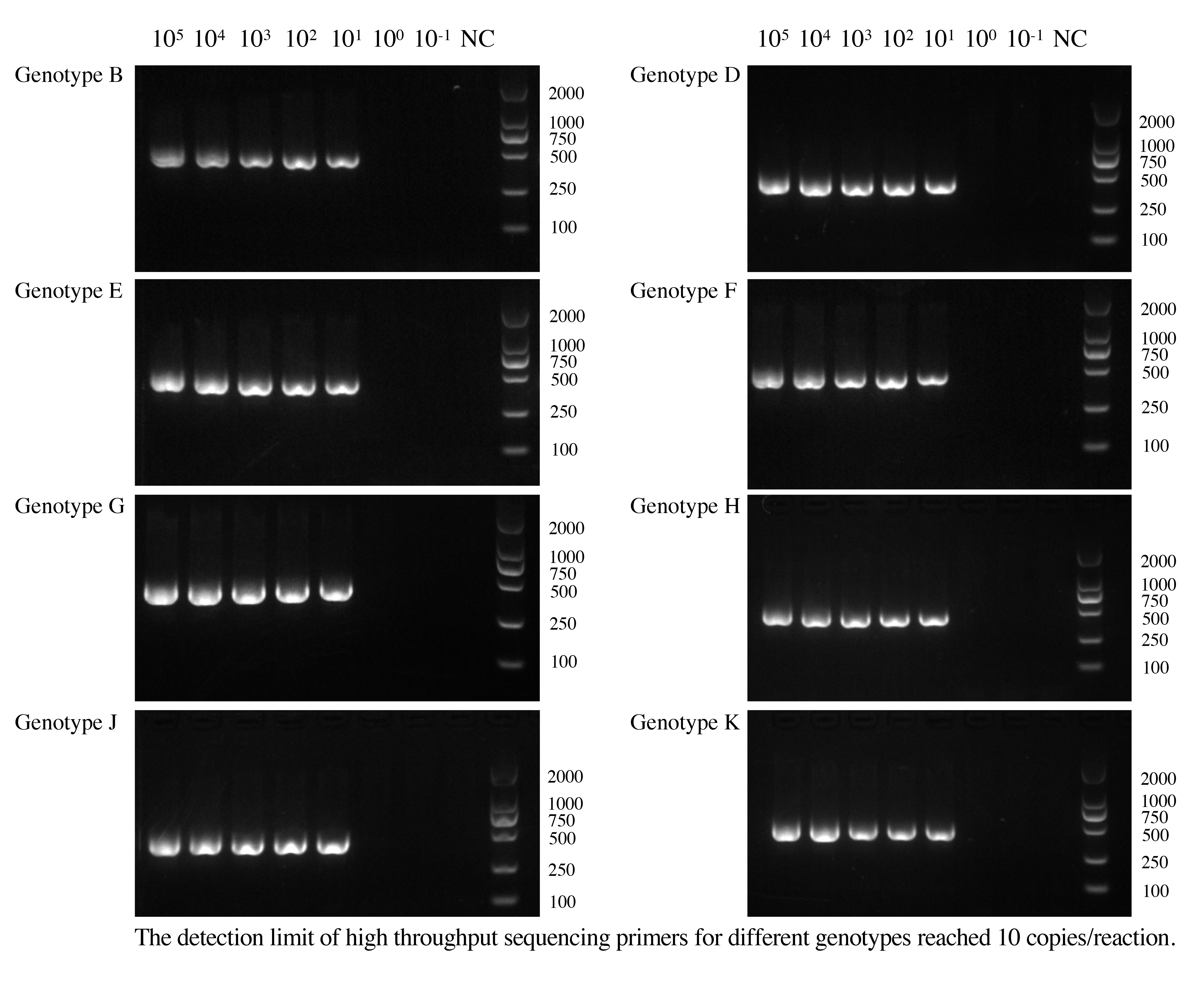

Supplement: SUPPLEMENTARY FIGURE S3 — Low detection limit of high-throughput sequencing primers for different Chlamydia trachomatis genotypes. [file Image_3.JPEG]

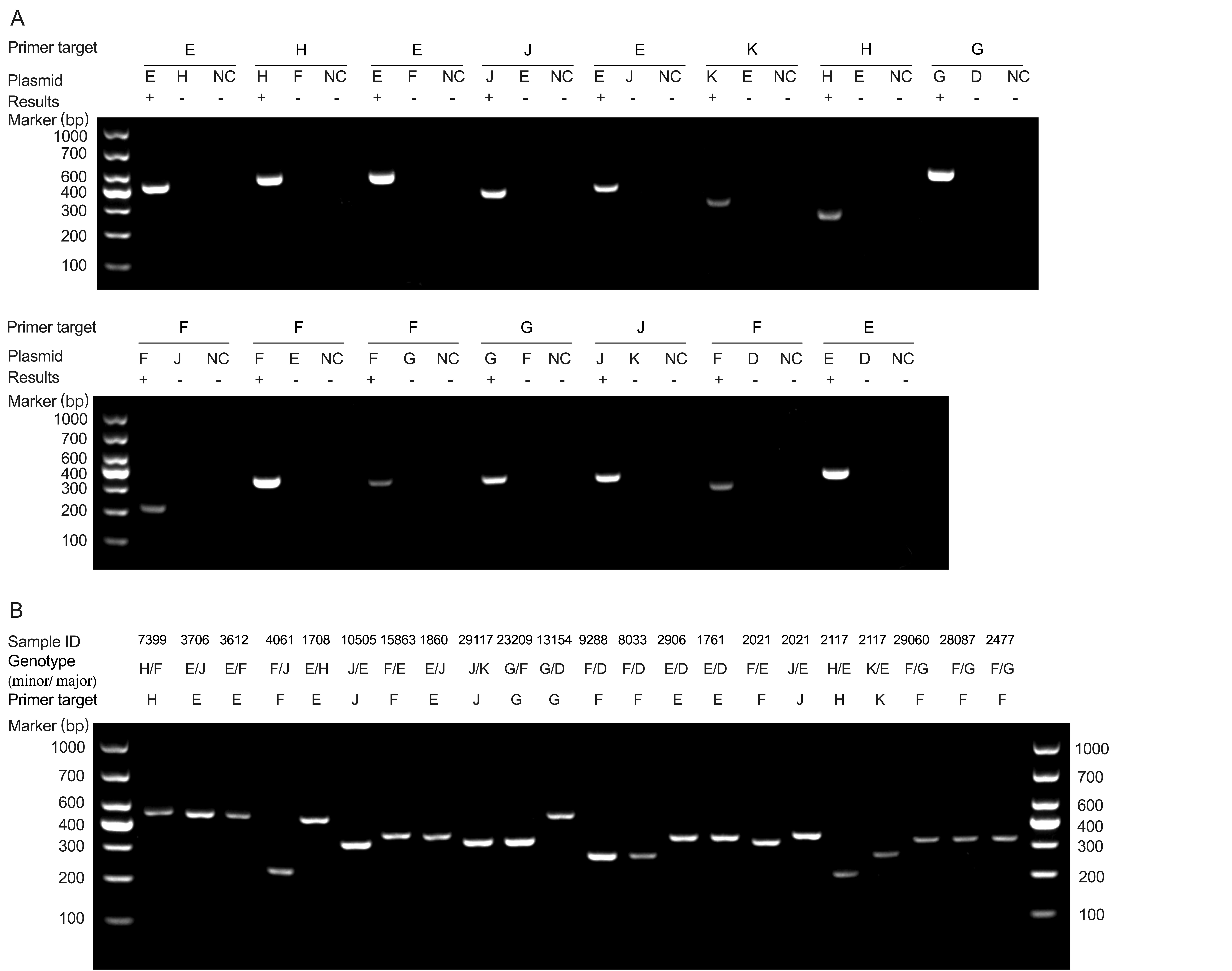

Supplement: SUPPLEMENTARY FIGURE S4 — Verification of genotype-specific primers for amplifying minor Chlamydia trachomatis (C. trachomatis) genotypes from mixed-genotype infections of C. trachomatis. (A) Genotype-specific primers can specifically amplify minor C. trachomatis genotypes when using plasmid DNAs of mixed-genotypes of C. trachomatis as templates. (B) The minor genotypes were specifically amplified from the samples of mixed-genotype infections by using genotype-specific primers. NC, negative control; +, PCR positive; -, PCR negative. [file Image_4.JPEG]
