# Supplementary material for: Identification and characterization of mixed infections of Chlamydia trachomatis via high-throughput sequencing
Source: Front Microbiol. 2022 Nov 10;13:1041789. doi: 10.3389/fmicb.2022.1041789 (PMC9687396; doi:10.3389/fmicb.2022.1041789)
Supplement: Supplementary file 5 [file Data_Sheet_1.docx]

## Supplementary Tables and Figures

**Table S1** Criteria for diagnosis of cervicitis and vagina cleanliness.

1. Diagnostic criteria of cervicitis

| **Clinical parameters** | **Score** | **Diagnosis criteria** |
| --- | --- | --- |
| Mucopurulent endocervical contents (swab test) | 3 | Cervicitis: score ≥ 3  No cervicitis: score ≤ 2 |
| Hypertrophy of cervical ectopy (ridged  or distorted the gross cervical architecture) | 3 |  |
| Friability on first swab | 1 |  |
| Erythema | 1 |  |
| Hypertrophy of cervical ectopy (protruded above the surrounding tissue) | 1 |  |

Ref: Batteiger, B., Lennington, W., Newhall, W., Katz, B., Morrison, H., and Jones, R. (1989).

1. Diagnostic criteria of vagina cleanliness

| **Vagina  cleanliness ^a^** | **Bacterium  vaginae ^b^** | **Coccus ^c^** | **Epithelial  Cell** | **Leukocytes/high power field** |
| --- | --- | --- | --- | --- |
| Ⅰ | ++++ | - | ++++ | 0～5 |
| Ⅱ | ++ | - | ++ | 5～15 |
| Ⅲ | - | ++ | - | 15～30 |
| Ⅳ | - | ++++ | - | >30 |

^a^ Class Ⅰ and Ⅱ were considered normal while class Ⅲ and Ⅳ as abnormal (Bao et al., 2015).

^b^ Bacterium vaginae mainly refers to Lactobacillus genus with five main species: L. crispatus, L. iners, L. jensenii, L. vaginalis and L. gasseri (Jespers et al., 2012).

^c^ Coccus mainly refers to Staphylococcus(Gajdács and Urbán, 2019), enterococcus(Sengupta et al., 2021), group B streptococcus(Vornhagen et al., 2018), Streptococcus agalactiae(Maniatis et al., 1996) and Neisseria gonorrhoeae(Silveira et al., 2020).

**Table S2** Information of primers used in this study.

| Primers | Sequence (5’-3’) | Size (bp) |
| --- | --- | --- |
| Detection primers ^a^ |  |  |
| CT-d-F | TTCCCCTTGTAATTCGTTGC | 20 |
| CT-d-R | TAGTAACTGCCACTTCATCA | 20 |
| Sanger sequencing primers |  |  |
| CT1 | CTCAACTGTAACTGCGTATTT | 21 |
| CT2 | CGGAAT TGTGCATTTACGTGAG | 23 |
| CT3 | TGAACCAAGCCTTATGATCGACGGA | 25 |
| CT4 | TCTTCGAYTTTAGGTTTAGATTGA | 24 |
| HTS primers |  |  |
| CT-HTS-F-outer | GTTTTCGACCGTGTTTTGAAAAC | 23 |
| CT-HTS-R-outer | AGGTTTAGATTGAGCGTATTGGAA | 24 |
| CT-HTS-F-inner | TTTGAAAACAGATGTGAATAAAGA | 24 |
| CT-HTS-R-inner | AGATTGAGCGTATTGGAAAGAAGC | 24 |
| HTS barcode-primers |  |  |
| CT-HTS-F-inner-B1 | **ATCACG**TTTGAAAACAGATGTGAATAAAGA | 30 |
| CT-HTS-F-inner-B2 | **CGATGT**TTTGAAAACAGATGTGAATAAAGA | 30 |
| CT-HTS-F-inner-B3 | **TTAGGC**TTTGAAAACAGATGTGAATAAAGA | 30 |
| CT-HTS-F-inner-B4 | **TGACCA**TTTGAAAACAGATGTGAATAAAGA | 30 |
| CT-HTS-F-inner-B5 | **ACAGTG**TTTGAAAACAGATGTGAATAAAGA | 30 |
| CT-HTS-F-inner-B6 | **GCCAAT**TTTGAAAACAGATGTGAATAAAGA | 30 |
| CT-HTS-F-inner-B7 | **CAGATC**TTTGAAAACAGATGTGAATAAAGA | 30 |
| CT-HTS-F-inner-B8 | **ACTTGA**TTTGAAAACAGATGTGAATAAAGA | 30 |
| CT-HTS-F-inner-B9 | **GATCAG**TTTGAAAACAGATGTGAATAAAGA | 30 |
| CT-HTS-F-inner-B10 | **TAGCTT**TTTGAAAACAGATGTGAATAAAGA | 30 |
| CT-HTS-R-inner-B25 | **ACTGAT**AGATTGAGCGTATTGGAAAGAAGC | 30 |
| CT-HTS-R-inner-B26 | **ATGAGC**AGATTGAGCGTATTGGAAAGAAGC | 30 |
| CT-HTS-R-inner-B27 | **ATTCCT**AGATTGAGCGTATTGGAAAGAAGC | 30 |
| CT-HTS-R-inner-B28 | **CAAAAG**AGATTGAGCGTATTGGAAAGAAGC | 30 |
| CT-HTS-R-inner-B29 | **CAACTA**AGATTGAGCGTATTGGAAAGAAGC | 30 |
| CT-HTS-R-inner-B30 | **CACCGG**AGATTGAGCGTATTGGAAAGAAGC | 30 |
| CT-HTS-R-inner-B31 | **CACGAT**AGATTGAGCGTATTGGAAAGAAGC | 30 |
| CT-HTS-R-inner-B32 | **CACTCA**AGATTGAGCGTATTGGAAAGAAGC | 30 |
| CT-HTS-R-inner-B33 | **CAGGCG**AGATTGAGCGTATTGGAAAGAAGC | 30 |
| CT-HTS-R-inner-B34 | **CATGGC**AGATTGAGCGTATTGGAAAGAAGC | 30 |

The reference sequences used in this study included A/Sa1(M58938), B/ IU1226 (AF063208), C/TW3 (M17343), D/ UW3 (AE001338), E/Bour (X52557), F/IC-Cal3 (X52080), G/UW57 (AF063199), H/UW4 (X16007), I/UW-12 (AF063200), J/UW36 (AF063202), K/UW31 (AF063204), L1/440 (M36533), L2/434 (M14738), and L3/404 (X55700). The bold sequence is the barcode sequence.

^a^ PCR and quantitative PCR were based on CT-d-F and CT-d-R primers.

**Table S3** Revalidation of mixed genotypes infection using genotype-specific primers.

| Sample ID | HTS  (Minor vs major genotype) | Genotype-specific primers ^a^ | | | | | Amplified fragment (bp) | Positive ^b^ |
| --- | --- | --- | --- | --- | --- | --- | --- | --- |
|  |  | Forward | Seq 5'-3' | | Reverse | Seq 5'-3' |  |  |
| 7399 | H vs F | F-2-subtype | | TGACAAGCCTACAAGTACTACAGGC | CT-HTS-R-inner | AGATTGAGCGTATTGGAAAGAAGC | 409 | YES |
| 3706 | E vs J | F-1-subtype | | AGCGGCGCCTACTACCAACGAT | CT-HTS-R-inner | AGATTGAGCGTATTGGAAAGAAGC | 400 | YES |
| 3612 | E vs F | F-3-subtype | | CAAGCCTACAAGTACTACAGGC | CT-HTS-R-inner | AGATTGAGCGTATTGGAAAGAAGC | 397 | YES |
| 4061 | F vs J | 4061F-F | | AATCCCGCTTATGGCAA | 4061F-R | CACCCACATTCCCAGAG | 213 | YES |
| 1708 | E vs H | F-1-subtype | | CTTTAGCCGGAGCTTCTGG | CT-HTS-R-inner | ACTATCTGCAGCAGGTT | 400 | YES |
| 10505 | J vs E | 2021J-F | | TGACAAGCCTACAAGTACTACAGGC | 2021J-R | AGATTGAGCGTATTGGAAAGAAGC | 305 | YES |
| 15863 | F vs E | 2021F-F | | GGAGCAACTACCGGTTATT | 2021F-R | ACCCACATTCCCAGAG | 334 | YES |
| 1860 | E vs J | 1860-E-F | | ACCAGCGATGTAGAAGG | 1860-E-R | CAAGATTCGCTGTATTAAACTT | 330 | YES |
| 29117 | J vs K | 29117J-F | | GCTTTAGCCGGAGCTTCTG | 29117J-R | AGATTGAGCGTATTGGAAAGAAGC | 310 | YES |
| 23209 | G vs F | CT-HTS-F-inner | | AGAGTTTGAAATGGGCGA | G/F-R1 | GAATTTCCTTTAAGATATCCACTG | 302 | YES |
| 13154 | G vs D | G/D-F2 | | CTTTAGCCGGAGCTTCTGG | CT-HTS-R-inner | ACTATCTGCAGCAGGTT | 399 | YES |
| 9288 | F vs D | F/D-F2 | | TTTGAAAACAGATGTGAATAAAGA | 8033F-R | GAATACTATCTGCAGCAGGTTT | 260 | YES |
| 8033 | F vs D | F/D-F2 | | TCGACCGTGTTTTGAAAACA | 8033F-R | GCTGTATTAAAGCTAGAAGC | 260 | YES |
| 2906 | E vs D | 2906E-F | | GGAGCTTCTGGGAATACGAC | E/D-R2 | CTGCACGAGCTCCAACA | 321 | YES |
| 1761 | E vs D | 2906E-F | | TTTGAAAACAGATGTGAATAAAGA | E/D-R2 | GAATACTATCTGCAGCAGGTTT | 321 | YES |
| 2021 | J vs E | 2021J-F | | AGTACTACAGGCAATGCTA | 2021J-R | GCCCACGCTCCAAGAG | 305 | YES |
| 2021 | F vs E | 2021F-F | | TTTGAAAACAGATGTGAATAAAGA | 2021F-R | TACTTGTTGCAGCAGGCTG | 334 | YES |
| 2117 | K vs E | 2117K-F | | AGTACTACAGGCAATGCTA | 2117K-R | GCCCACGCTCCAAGAG | 259 | YES |
| 2117 | H vs E | 2117H-F | | TTTGAAAACAGATGTGAATAAAGA | 2117H-R | GAATACTATCTGCAGCAGGTTT | 202 | YES |
| 29060 | F vs G | CT-HTS-F-inner | | AATCCCGCTTATGGCAA | F/G-R1 | CACCCACATTCCCAGAG | 305 | YES |
| 28087 | F vs G | CT-HTS-F-inner | | GGAGCTTCTGGGAATACGAC | F/G-R1 | CTGCACGAGCTCCAACA | 305 | YES |
| 2477 | F vs G | CT-HTS-F-inner | | CAGCTCCAACCACTCTTAC | F/G-R1 | ATCCGCACTCCCACAA | 305 | YES |

^a^ For each sample with mixed-genotype infections, the genotype-specific primer was designed to match the sequence of minor genotype but not the major genotype, especially in the 3’ end.

^b^ Positive results mean that the minor genotypes were successfully amplified by the corresponding specific primers.

**Table S4** Genotype distribution of *C. trachomatis* in 20 specimens of mixed-genotype infections using next generation high-throughput sequencing (NGHTS) and Sanger sequencing of individual clones from Chenzhou, China during 2019 and 2021.

| Sample ID | *C. trachomatis* genotypes of NGHTS (reads %) | | | | *C. trachomatis* genotypes of Sanger sequence (N %) | |
| --- | --- | --- | --- | --- | --- | --- |
|  | First test | | Second test | | Major genotype | Minor genotype |
|  | Major genotype | Minor genotype | Major genotype | Minor genotype |  |  |
| CZ-13154 | D 1495589 (52.19) | G 1369971 (47.81) |  |  |  |  |
| CZ-2906 | D 57498 (53.40) | E 50172 (46.60) |  |  |  |  |
| CZ-7399 | F 1267351 (59.95) | H 838830 (39.68) | F 186157 (67.81) | H 90452 (32.56) | F 14 (93.33) | H 1 (6.67) |
| CZ-3706 | J 1266951 (60.90) | E 804196 (38.66) | J 236444 (67.81) | E 112165 (32.17) | J 13 (72.22) | E 5 (27.28) |
| CZ-8033 | D 1023163 (62.74) | F 599935 (36.79) | F 122705 (78.15) | D 84736 (40.83) | D 7 (50.00) | F 7 (50.00) |
| CZ-9288 | D 90742 (69.65) | F 39218 (30.10) |  |  |  |  |
| CZ-3612 | F 1235996 (72.91) | E 458921 (27.07) | F 100409 (78.15) | E 27803 (21.64) | F 14 (93.33) | E 1 (50.00) |
| CZ-2117 | E 882877 (81.12) | H 185179 (17.01);  K 14015 (1.29) |  |  | H 14 (77.78) | E 4 (22.22) |
| CZ-2021 | E 161181 (84.39) | J 26375 (13.81);  F 3116 (1.63) | E 206250 (84.12) | J 34666 (14.14);  F 3585 (1.46%) |  |  |
| CZ-4061 | J 1996995 (89.06) | F 245339 (10.94) | J 192964 (65.76) | F 100242 (34.16) |  |  |
| CZ-1761 | D 116786 (93.57) | E 8027 (6.43) |  |  |  |  |
| CZ-29060 | G 77444 (97.51) | F 1244 (6.43) |  |  |  |  |
| CZ-1708 | H 1848791 (97.56) | E 39242 (2.07) |  |  | H 28 (100.00) | 0 |
| CZ-10505 | E 1690609 (97.73) | J 26740 (1.55) |  |  | E 23 (100.00) | 0 |
| CZ-1860 | J 142371 (98.05) | E 2829 (1.95) |  |  |  |  |
| CZ-23209 | F (98.22) 193812 | G 3480 (1.76) |  |  |  |  |
| CZ-2477 | G (98.28) 137418 | F 1486 (1.06) |  |  |  |  |
| CZ-29117 | K (98.46) 98083 | J 1521 (1.53) |  |  |  |  |
| CZ-28087 | G 275295 (98.68) | F 3574 (1.28) |  |  |  |  |
| CZ-15863 | E 60889 (98.76) | F 757 (1.23) |  |  |  |  |

**Table S5** Comparation of the genotyping result in both Sanger sequencing and Next-generation high-throughput sequencing (NGHTS).

| Genotypes of *C. trachomatis* | Methods | | *P-*value ^a^ |
| --- | --- | --- | --- |
|  | Sanger sequencing | NGHTS |  |
| B | 2 | 2 | >0.999 |
| D | 40 | 40 |  |
| E | 83 | 83 |  |
| F | 65 | 65 |  |
| G | 18 | 18 |  |
| H | 10 | 10 |  |
| J | 55 | 55 |  |
| K | 10 | 10 |  |

**^a^** *P*-value was calculated by McNemar test.

**Table S6** Relationship of selected demographic and epidemiological data to *C. trachomatis* genotypes in 303 *C. trachomatis*-infected women from Chenzhou, China, 2019 to 2021.

| **Characteristics** | % Of Genotype (n=261) ^a^ | | | | | *P*  value | % Of Genotype group (n=283) | | | *P*  value | % Of Single  n=283 | % Of Mixed ^b^ n=20 | *P* ^c^ value |
| --- | --- | --- | --- | --- | --- | --- | --- | --- | --- | --- | --- | --- | --- |
|  | D n=40 | E n=83 | F n=65 | G n=18 | J n=55 |  | B-complex n=125 | F/G group n=83 | C-complex n=75 |  |  |  |  |
| **Age (year)** |  |  |  |  |  |  |  |  |  |  |  |  |  |
| ≤25 | 17.5 | 21.7 | 20.0 | 27.8 | 20.0 | 0.610 | 20.0 | 21.7 | 21.3 | 0.656 | 20.8 | 20.0 | 0.343 |
| 25-35 | 55.0 | 45.8 | 43.1 | 44.4 | 49.1 |  | 48.0 | 43.4 | 49.3 |  | 47.0 | 35.0 |  |
| 35-45 | 15.0 | 22.9 | 15.4 | 16.7 | 18.2 |  | 20.0 | 15.7 | 17.3 |  | 18.0 | 35.0 |  |
| >45 | 12.5 | 9.6 | 21.5 | 11.1 | 12.7 |  | 12.0 | 19.3 | 12.0 |  | 14.1 | 10.0 |  |
| **Clinical departments ^d^** |  |  |  |  |  |  |  |  |  |  |  |  |  |
| PEC | 35.0 | 21.7 | 32.3 | 38.9 | 20.0 | 0.349 | 26.4 | 33.7 | 20.0 | 0.268 | 26.9 | 20.0 | 0.449 |
| ART | 12.5 | 16.9 | 20.0 | 22.2 | 25.5 |  | 15.2 | 20.5 | 22.7 |  | 18.7 | 30.0 |  |
| GC | 52.5 | 61.4 | 47.7 | 38.9 | 54.5 |  | 58.4 | 45.8 | 57.3 |  | 54.4 | 50.0 |  |
| **Antibiotic usage (previous 3 months)** | | | | | | | | | | | | | |
| Yes | 10.0 | 2.4 | 3.1 | 5.6 | 5.5 | 0.401 | 4.8 | 3.6 | 6.7 | 0.597 | 4.9 | 0.0 | 0.61 |
| No | 90.0 | 97.6 | 96.9 | 94.4 | 94.5 |  | 95.2 | 96.4 | 93.3 |  | 95.1 | 100.0 |  |
| **HPV infection ^e^** |  |  |  |  |  |  |  |  |  |  |  |  |  |
| No | 65.0 | 78.3 | 80.0 | 88.9 | 76.4 | 0.603 | 72.8 | 81.9 | 77.3 | 0.729 | 76.7 | 75.0 | 0.854 |
| Low-risk genotypes | 2.5 | 3.6 | 1.5 | 0.0 | 1.8 |  | 3.2 | 1.2 | 1.3 |  | 2.1 | 0.0 |  |
| High-risk genotypes | 32.5 | 18.1 | 18.5 | 11.1 | 21.8 |  | 24.0 | 16.9 | 21.3 |  | 21.2 | 25.0 |  |

^a^ Genotypes of <5% of the total patients were included in the corresponding genogroups for analysis.

^b^ Mixed *C. trachomatis* genotypes infection

^c^ P values were calculated using Chi-square tests.

^d^ PEC, Physical Examination Center; ART, Assisted Reproductive Technology; GC, Gynecology Clinics.

^e^ Low risk genotypes of human papillomavirus (HPV) include HPV 6, 11 and CP8304 while high-risk HPV genotypes include HPV 16, 18, 31, 33, 39, 45, 51, 52, 53, 56, 58, 59, 66, and 68.

**Table S7** Symptoms of *C. trachomatis* positive women from Chenzhou, China during 2019 and 2021.

| **Symptoms** | ***C. trachomatis* infection (n, %)** | | | ***P* ^a^**  **value** |
| --- | --- | --- | --- | --- |
|  | **Total n=311** | **Genotype defined n=303** | **Genotype undefined n=8** |  |
| Asymptomatic ^b^ | 93 (29.9) | 88 (29.0) | 5 (62.5) | 0.099 |
| Vaginosis | 119 (38.3) | 117 (38.6) | 2 (25.0) | 0.679 |
| Pelvic Inflammatory Disease ^c^ | 34 (10.9) | 34 (11.2) | 0 | 0.605 |
| Cervicitis | 26 (8.4) | 26 (8.6) | 0 | >0.999 |
| Irregular menstruation | 24 (7.7) | 24 (7.7) | 0 | >0.999 |
| Intermenstrual bleeding | 19 (6.1) | 19 (6.3) | 0 | >0.999 |
| Contact bleeding | 12 (3.9) | 12 (4.0) | 0 | >0.999 |
| Endometritis | 7 (2.3) | 7 (2.3) | 0 | >0.999 |
| Lower abdominal pain | 7 (2.3) | 7 (2.3) | 0 | >0.999 |
| Ovarian cyst | 5 (1.6) | 5 (1.7) | 0 | >0.999 |
| Urethritis | 4 (1.3) | 4 (1.3) | 0 | >0.999 |
| Pointed condyloma | 3 (1.0) | 3 (1.0) | 0 | >0.999 |
| Hysteromyoma | 2 (0.6) | 2 (0.7) | 0 | >0.999 |
| Ectopic pregnancy | 2 (0.6) | 2 (0.7) | 0 | >0.999 |
| Spontaneous abortion | 1 (0.3) | 1 (0.3) | 0 | >0.999 |

^a^ *P* values were calculated using Chi-square tests.

^b^ Asymptomatic C. trachomatis infection was defined as positive for C. trachomatis nucleic acid without symptoms, such as painful sexual intercourse, abnormal vaginal discharge, urethritis, irregular vaginal bleeding, or bleeding after sexual intercourse and genital warts (Chen et al., 2020).

^c^ Pelvic inflammatory disease (PID) was defined as tenderness with adnexal, cervical motion, and uterine tenderness (Dean et al., 1995).

**Table S8** Comparison of clinical manifestations in women infected with *C. trachomatis* genotype G to those infected with genotype E.

| **Clinical Manifestations** | ***C. trachomatis* single genotype infection** | | | |
| --- | --- | --- | --- | --- |
|  | **Genotype E (N=83) ^a^ event /N (%)** | **Genotype G (N=18)** | | |
|  |  | **event /N (%)** | **OR (95% CI) ^b^** | **aOR (95% CI) ^c^** |
| **Asymptomatic** | 22/83 (26.5) | 6/18 (33.3) | 0.69 (0.18 - 2.67) | 1.58 (0.51 - 4.87) |
| **Vaginosis** | 33/83 (39.8) | 5/18 (27.8) | 0.73 (0.22 - 2.42) | 0.6 (0.2 -1.86) |
| **Pelvic Inflammatory Disease** | 8/83 (9.6) | 5/18 (27.8) | **6.06 (1.29 - 28.5)** | **3.61 (1.02 - 12.8)** |
| **Cervicitis** | 5/83 (6.00) | 2/18 (11.1) | 1.88 (0.32 - 10.9) | 1.97 (0.35 - 11.1) |
| **Vagina cleanliness (vs. Ⅰ/Ⅱ)** | | | | |
| **Ⅲ** | 16/55 (29.1) | 6/15 (40.0) | 3.16 (0.65 - 15.32) | 3.0 (0.64 - 14.01) |
| **Ⅳ** | 15/55 (27.3) | 6/15 (40.0) | **6.91 (1.25 - 38.09)** | 3.0 (0.65 - 13.94) |
| **Cervical abnormalities (vs. Benign) ^d^** | | | | |
| ASC-US | 4/67 (6.0) | 0 | NA ^e^ | NA |
| SIL | 4/67 (6.0) | 1/15 (6.7) | 1.15 (0.11 - 11.98) | 1.08 (0.11 - 10.6) |

^a^ Genotype E was set as reference group.

^b^ Odds ratio (OR) was calculated according to multivariate logistic regression analysis by controlling age, clinical departments, antibiotic usage and HPV infection.

^c^ Adjusted odd ratio (aOR) was calculated based on the data with propensity score weighting. Bold OR and aOR indicate statistically significant, i.e. *P*<0.05.

^d^ ASC-US, atypical squamous cells of undetermined significance; SIL, squamous intraepithelial lesion.

^e^ NA, not applicable.

**Table S9** Demographic and epidemiological characteristics of *C. trachomatis* genotype E and G infections after propensity score weighting ^a^.

| **Characteristics** | ***C. trachomatis* single genotype infection (n, %)** | | |
| --- | --- | --- | --- |
|  | **Genotype G n=18** | **Genotype E n=83** | ***P* ^b^  value** |
| **Age (Mean ± SE）** | 32.4 ± 2.1 | 32.3 ±2.4 | 0.987 |
| **Clinical departments ^c^** |  |  |  |
| PEC | 7 (38.9) | 32 (38.6) | 0.998 |
| ART | 4 (22.2) | 19 (22.9) |  |
| GC | 7 (38.9) | 32 (38.6) |  |
| **Antibiotic usage (previous 3 months)** |  |  |  |
| Yes | 1 (5.6) | 6 (7.2) | >0.999 |
| No | 17 (94.4) | 77 (92.8) |  |
| **HPV infection** |  |  |  |
| Yes | 2 (11.1) | 10 (12.0) | >0.999 |
| No | 16 (88.9) | 73 (88.0) |  |

^a^ Propensity score of genotype G (cases) was generated based on multivariate binary logistic regression analysis by controlling age, clinical departments, antibiotic usage and HPV infection, and further adjusted by standardized mortality ratio weighting (SMRW) methods.

^b^ *P* values were calculated using Chi-square tests.

^c^ PEC, Physical Examination Center; ART, Assisted Reproductive Technology; GC, Gynecology Clinics.

**Table S10** Demographic and epidemiological characteristics of *C. trachomatis* single- or mixed-genotype infections after propensity score weighting ^a^.

| **Characteristics** | ***C. trachomatis* infection (n, %)** | | | | | | | | |
| --- | --- | --- | --- | --- | --- | --- | --- | --- | --- |
|  | **Total mixed n=20** | **Single n=282** | ***P* ^b^  value** | **With dominant ^c^ genotype n=10** | **Single n=282** | ***P*  value** | **Without dominant genotype n=10** | **Single n=290** | ***P*  value** |
| **Age**  **(Mean ± SE）** | 35.2 ± 1.97 | 35.3 ± 2.17 | 0.981 | 34.9 ± 2.24 | 34.8 ± 3.79 | 0.996 | 35.5 ± 3.37 | 36.9 ± 4.66 | 0.956 |
| **Clinical departments ^d^** | | | | | | | | | |
| PEC | 4 (20.0) | 58 (20.6) | >0.999 | 4 (40.0) | 116 (41.1) | >0.999 | 0 | 0 | >0.999 |
| ART | 6 (30.0) | 80 (28.4) |  | 4 (40.0) | 109 (38.7) |  | 2 (20.0) | 54 (18.6) |  |
| GC | 10 (50.0) | 144 (51.1) |  | 2 (20.0) | 57 (20.2) |  | 8 (80.0) | 236 (81.4) |  |
| **Antibiotic usage (previous 3 months)** | | | | | | | | | |
| Yes | 0 | 0 | >0.999 | 0 | 0 | >0.999 | 0 | 0 | >0.999 |
| No | 20 (100.0) | 282 (100.0) |  | 10 (100.0) | 282 (100.0) |  | 10 (100.0) | 290 (100.0) |  |
| **HPV infection** | | | | | | | | | |
| Yes | 5 (25.0) | 70 (24.8) | >0.999 | 3 (30.0) | 79 (28.0) | >0.999 | 2 (20.0) | 60 (20.7) | >0.999 |
| No | 15 (75.0) | 212 (75.2) |  | 7 (70.0) | 203 (72.0) |  | 8 (80.0) | 230 (79.3) |  |
| **CT genotype (major / single)** | | | | | | | | | |
| D | 5 (25.0) | 68 (24.1) | >0.999 | 1 (10.0) | 29 (10.3) | >0.999 | 4 (40.0) | 122 (42.1) | >0.999 |
| E | 4 (20.0) | 57 (20.2) |  | 2 (20.0) | 58 (20.6) |  | 2 (20.0) | 56 (19.3) |  |
| F | 3 (15.0) | 42 (14.9) |  | 1 (10.0) | 28 (9.9) |  | 2 (20.0) | 55 (19.0) |  |
| G | 3 (15.0) | 43 (15.2) |  | 3 (30.0) | 83 (29.4) |  | 0 | 0 |  |
| H | 1 (5.0) | 15 (5.3) |  | 1 (10.0) | 30 (10.6) |  | 0 | 0 |  |
| J | 3 (15.0) | 44 (15.6) |  | 1 (10.0) | 28 (9.9) |  | 2 (20.0) | 57 (19.7) |  |
| K | 1 (5.0) | 13 (4.6) |  | 1 (10.0) | 26 (9.2) |  | 0 | 0 |  |

^a^ Propensity scores for mixed-genotype infections with or without dominant genotype were generated based on multivariate binary logistic regression analysis by controlling age, clinical departments, antibiotic usage, HPV infection and *C. trachomatis* genotypes. The scores were further adjusted by standardized mortality ratio weighting (SMRW) methods.

^b^ *P* values were calculated using Chi-square tests.

^c^ Mixed-genotype infections were divided into groups with or without dominant genotypes according the proportion of minor genotypes <10% or ≥10%.

^d^ PEC, Physical Examination Center; ART, Assisted Reproductive Technology; GC, Gynecology Clinics.

**Reference**

Bao, Y., Hu, M., Gao, G., Huang, J., and Zhang, Z. (2015). [Multivariate analysis for pelvic floor dysfunction]. *Zhong Nan Da Xue Xue Bao Yi Xue Ban* 40(11)**,** 1229-1233. doi: 10.11817/j.issn.1672-7347.2015.11.011.

Chen, H., Luo, L., Wen, Y., He, B., Ling, H., Shui, J., et al. (2020). Chlamydia trachomatis and Human Papillomavirus Infection in Women From Southern Hunan Province in China: A Large Observational Study. *Frontiers in microbiology* 11**,** 827. doi: 10.3389/fmicb.2020.00827.

Dean, D., Oudens, E., Bolan, G., Padian, N., and Schachter, J. (1995). Major outer membrane protein variants of Chlamydia trachomatis are associated with severe upper genital tract infections and histopathology in San Francisco. *The Journal of infectious diseases* 172(4)**,** 1013-1022. doi: 10.1093/infdis/172.4.1013.

Gajdács, M., and Urbán, E. (2019). Epidemiology and resistance trends of Staphylococcus aureus isolated from vaginal samples: a 10-year retrospective study in Hungary. *Acta Dermatovenerol Alp Pannonica Adriat* 28(4)**,** 143-147.

Jespers, V., Menten, J., Smet, H., Poradosú, S., Abdellati, S., Verhelst, R., et al. (2012). Quantification of bacterial species of the vaginal microbiome in different groups of women, using nucleic acid amplification tests. *BMC Microbiol* 12**,** 83. doi: 10.1186/1471-2180-12-83.

Maniatis, A.N., Palermos, J., Kantzanou, M., Maniatis, N.A., Christodoulou, C., and Legakis, N.J. (1996). Streptococcus agalactiae: a vaginal pathogen? *J Med Microbiol* 44(3)**,** 199-202. doi: 10.1099/00222615-44-3-199.

Sengupta, M., Sarkar, S., SenGupta, M., Ghosh, S., Sarkar, R., and Banerjee, P. (2021). Biofilm Producing Enterococcus Isolates from Vaginal Microbiota. *Antibiotics (Basel)* 10(9). doi: 10.3390/antibiotics10091082.

Silveira, M.F., Bruni, M.P., Stauffert, D., Golparian, D., and Unemo, M. (2020). Prevalence and risk factors associated with Chlamydia trachomatis, Neisseria gonorrhoeae, and Mycoplasma genitalium among women in Pelotas, Southern Brazil. *Int J STD AIDS* 31(5)**,** 432-439. doi: 10.1177/0956462419898982.

Vornhagen, J., Armistead, B., Santana-Ufret, V., Gendrin, C., Merillat, S., Coleman, M., et al. (2018). Group B streptococcus exploits vaginal epithelial exfoliation for ascending infection. *J Clin Invest* 128(5)**,** 1985-1999. doi: 10.1172/jci97043.
